# China-US grain trade shapes the spatial genetic pattern of common ragweed in East China cities

**DOI:** 10.1038/s42003-023-05434-5

**Published:** 2023-10-21

**Authors:** Siran Lu, Xiangyu Luo, Hongfang Wang, Rodolfo Gentili, Sandra Citterio, Jingyi Yang, Jing Jin, Jianguang Li, Jun Yang

**Affiliations:** 1 Department of Earth System Science, Institute for Global Change Studies, Ministry of Education Ecological Field Station for East Asian Migratory Birds, Tsinghua University, Beijing, 100084 China; 2 Sichuan Forestry and Grassland Bureau, Chengdu, 610081 China; 3 College of Life Sciences, Beijing Normal University, Beijing, 100875 China; 4 Department of Earth and Environmental Sciences, University of Milan-Bicocca, Piazza della Scienza 1, I-20126 Milan, Italy; 5 College of Forestry, Guizhou University, Guiyang, 550025 China; 6 Information Center of Jiangsu Academy of Agricultural Sciences, Nanjing, 210014 China; 7 Beijing Customs District P. R. China, Beijing, 100026 China; 8 Present Address: Ministry of Education Key Laboratory for Biodiversity Science and Ecological Engineering, National Observations and Research Station for Wetland Ecosystems of the Yangtze Estuary, Institute of Biodiversity Science and Institute of Eco-Chongming, School of Life Sciences, Fudan University, Shanghai, 200438 China

**Keywords:** Ecological genetics, Invasive species, Urban ecology

## Abstract

Common ragweed is an invasive alien species causing severe allergies in urban residents. Understanding its urban invasion pathways is crucial for effective control. However, knowledge is limited, with most studies focusing on agricultural and natural areas, and occurrence record-based studies exhibiting uncertainties. We address this gap through a study in East China cities, combining population genetics and occurrence records. Leaf samples from 37 urban common ragweed populations across 15 cities are collected. Genomic and chloroplast DNA extraction facilitate analysis of spatial genetic patterns and gene flows. Additionally, international grain trade data is examined to trace invasion sources. Results indicate spatial genetic patterns impacted by multiple introductions over time. We infer the modern grain trade between the United States and China as the primary invasion pathway. Also, cities act as transportation hubs and ports of grain importation might disperse common ragweed to urban areas. Invasive species control should account for cities as potential landing and spread hubs of common ragweed.

## Abstract (web summary)

Examination of the spatial genetic pattern of common ragweed plants in urban areas in East China cities and the influence of grain trade between China and the U.S suggestions multiple invasion events.

## Introduction

Common ragweed (*Ambrosia artemisiifolia* L., Asteraceae) is native to North America but is now an invasive alien species (IAS) on all continents except Antarctica^1^. Besides causing significant losses in agricultural production, common ragweed pollen is a primary allergen source, causing seasonal allergic rhinitis and asthma, which result in substantial health costs and labor losses^1,2^. The negative health impact of common ragweed pollens is worsened in urban areas because the production of pollens and allergenicity of pollens are both enhanced in urban environments^3,4^. Therefore, control of the common ragweed is a priority in many cities worldwide^5^.

Cities need information on the invasion pathway of common ragweed to control it more effectively. So far, this information has been primarily inferred from occurrence records. For example, the introduction of common ragweed was speculated to be related to grain shipment based on surveying the distribution of common ragweed in Israel^6^. In Magdeburg, Germany, researchers used the occurrence records of common ragweed populations in the urban area to link the introduction of common ragweed to seed trade^7^. Based on the occurrence data, communication lines were found to play a vital role in the dispersal of common ragweed in urban areas of Croatia^8^. While those inferences are helpful, they also contain notable limitations. The study in Israel and Magdeburg could not further trace the source of common ragweed to countries exporting the contaminated seeds^7^. It is difficult to determine the real dispersal hub of common ragweed in urban areas of Croatia^8^.

Some typical features of occurrence records contributed to the limitations in the above studies. First, like many other IAS, the data quality and availability of occurrence records for common ragweed are often inadequate^9,10^. The quality and availability of occurrence data for IAS in cities are particularly uneven^10,11^. Records with spatial and temporal errors may lead to an incomplete or even wrong interpretation of invasion pathways in the IAS. Second, interpretation of the occurrence data is challenging even when the quality of the data is not an issue. The spatial distribution of a species is not always a good indication of its spread pathway. For instance, plant populations with distinct ancestors in a city may be incorrectly judged as close relatives due to their spatial proximity^12^.

Population genetic methods provide a viable way to address the above limitations. The phylogenetic relationship among populations of IAS can be reconstructed using various genetic methods to interpret the invasion pathway. For example, in Argentina, the genetic analysis showed that the common sunflower (*Helianthus annuus* L.) was first introduced to Buenos Aires and then spread to Mendoza^13^. The population structure of slender false brome (*Brachypodium sylvaticum* (Huds.) P. Beauv.) in Corvallis, the United States, showed that the species was introduced from multiple locations in Europe^14^. Studies have already shown the potential of population genetic methods for revealing the invasion pathways of common ragweed. The method has been used to trace the source of common ragweed populations in Asia, South America, and Western and Eastern Europe^15–17^. It has also been adopted to identify the invasion pathways in Australia^18^ and quantify the genetic patterns of common ragweed populations in North America and China^19,20^. The invasion sources of common ragweed populations in East China was explored in an earlier study^21^. However, several key questions have not been answered. How did the propagules of common ragweed enter China? Are invasion sources different for common ragweed populations in East China cities? Which city(cities) is the dispersal centers of common ragweed? The answers to the above questions are crucial for devising effective and targeted management measures for common ragweed.

In this study, we intend to disclose the invasion pathways of common ragweed in urban areas. We selected cities in East China as the sites for this case study. China first recorded common ragweed in the 1930s^22^. Common ragweed currently occurs in more than 23 provinces, and most of the occurrences are in East China^23,24^. East China accounts for 43% of China’s land area but nearly 94% of China’s population^25^. The highly urbanized region contains key port cities for international trade and a well-developed transportation network^26^. Because existing studies found that ragweed seed is a common contaminant of the seed trade and international trade can accelerate the introduction of common ragweed^27,28^, we hypothesized that the international trade would contribute to the introduction of common ragweed into urban areas in East China cities. To test this hypothesis, we employed a combination of population genetic data, occurrence records, and international grain trade data. Our findings added new evidence of the facilitating role of international trade in spreading common ragweed.

## Results

### Genetic patterns of common ragweed populations in East China cities

We identified six nuclear microsatellite loci from the leaf samples. The results of exact tests for genotypic disequilibrium showed that none of the microsatellite loci presented significant linkage equilibrium. Also, the estimates of null alleles were highly variable (Supplementary Fig. 1). Therefore, these loci could be used for further analysis. Deviation from Hardy-Weinberg proportions was detected in most common ragweed populations (Table 1), which suggested heterozygote deficiency. Bottleneck effects caused by the population size reduction were found in six populations.

**Table 1:** Summary statistics of the genetic diversity of the common ragweed populations in East China cities.

Cities	Population ID	n1/n2	N_A_	H_E_	F_IS_	*h*	H_D_	π
Beijing	BJ1	13/12	4.50	0.650	0.074	1	0	0
Beijing	BJ2	15/13	5.33	0.644	**0.341**	1	0	0
Beijing	BJ3	16/13	6.17	0.726	**0.494**	1	0	0
Beijing	BJ4	16/13	6.67	0.728	**0.351**	2	0.52	0.0007
Changchun	CC1	10/10	7.00	0.732	**0.303**	1	0	0
Changchun	CC4	11/9	7.83	0.774	**0.329**	1	0	0
Changchun	CC6	10/10	7.83	0.709	**0.301**	1	0	0
Chongqing	CQ2	8/7	4.00	0.607	**0.515**	2	0.26	0.001
Changsha	CS1	6/1	5.00	0.694	**0.324**	1	0	0
Changsha	CS2	13/0	6.00	0.677	**0.243**	na	na	na
Fushun	FS1*	15/14	6.00	0.667	**0.472**	1	0	0
Fushun	FS2	14/14	9.00	0.751	**0.399**	3	0.46	0.0007
Fushun	FS3*	9/9	4.33	0.501	**0.389**	1	0	0
Fushun	FS4	15/12	8.33	0.730	**0.384**	2	0.16	0.0002
Fuzhou	FZ3	15/10	8.33	0.804	**0.436**	2	0.19	0
Fuzhou	FZ4	11/8	7.00	0.750	**0.469**	2	0.53	0.0007
Fuzhou	FZ5	8/8	6.83	0.777	**0.369**	1	0	0
Guiyang	GY1	15/12	4.83	0.613	**0.511**	1	0	0
Guangzhou	GZ1	10/9	6.50	0.753	**0.162**	2	0.47	0.0012
Mudanjiang	MDJ1	16/13	8.67	0.751	**0.314**	2	0.41	0.0005
Mudanjiang	MDJ2	16/14	9.17	0.811	**0.387**	3	0.37	0.0005
Mudanjiang	MDJ3	15/14	7.83	0.766	**0.421**	3	0.37	0.0005
Mudanjiang	MDJ4*	15/12	7.67	0.784	**0.285**	3	0.68	0.0011
Nanjing	NJ1	19/18	10.33	0.823	**0.493**	3	0.55	0.0008
Nanjing	NJ4	13/13	7.83	0.761	**0.471**	3	0.57	0.0008
Nanjing	NJ5	9/8	5.50	0.685	**0.355**	3	0.63	0.001
Qingdao	QD1	12/3	5.50	0.729	**0.268**	1	0	0
Qingdao	QD2*	11/7	7.17	0.782	**0.365**	1	0	0
Qingdao	QD4	14/6	7.17	0.771	**0.327**	1	0	0
Qinhuangdao	QHD1*	10/4	7.33	0.747	**0.355**	2	0.57	0.0007
Qinhuangdao	QHD2	11/11	8.00	0.766	**0.356**	2	0.17	0.0002
Qinhuangdao	QHD3	12/9	8.83	0.802	**0.362**	2	0.47	0.0006
Shanghai	SH1*	7/6	3.33	0.537	0.189	1	0	0
Shenyang	SY1	15/13	5.83	0.607	**0.365**	1	0	0
Shenyang	SY2	9/8	4.00	0.541	−0.198	1	0	0
Shenyang	SY3	15/15	6.83	0.646	**0.207**	1	0	0
Wuhan	WH2	17/16	9.83	0.791	**0.449**	2	0.39	0.0005

Bold values of F_IS_ indicate significant *(p* < 0.05) deviations from Hardy-Weinberg equilibrium. An asterisk next to the population ID indicates an experienced bottleneck effect. n1, number of individuals sampled. N_.A._, number of different alleles. H_E_, expected heterozygosity. F_IS_, inbreeding coefficient. n2, number of individuals used for cpDNA analysis. *h*, number of haplotypes. H_.D._, haplotype diversity. π, nucleotide diversity.

**Fig. 1:**
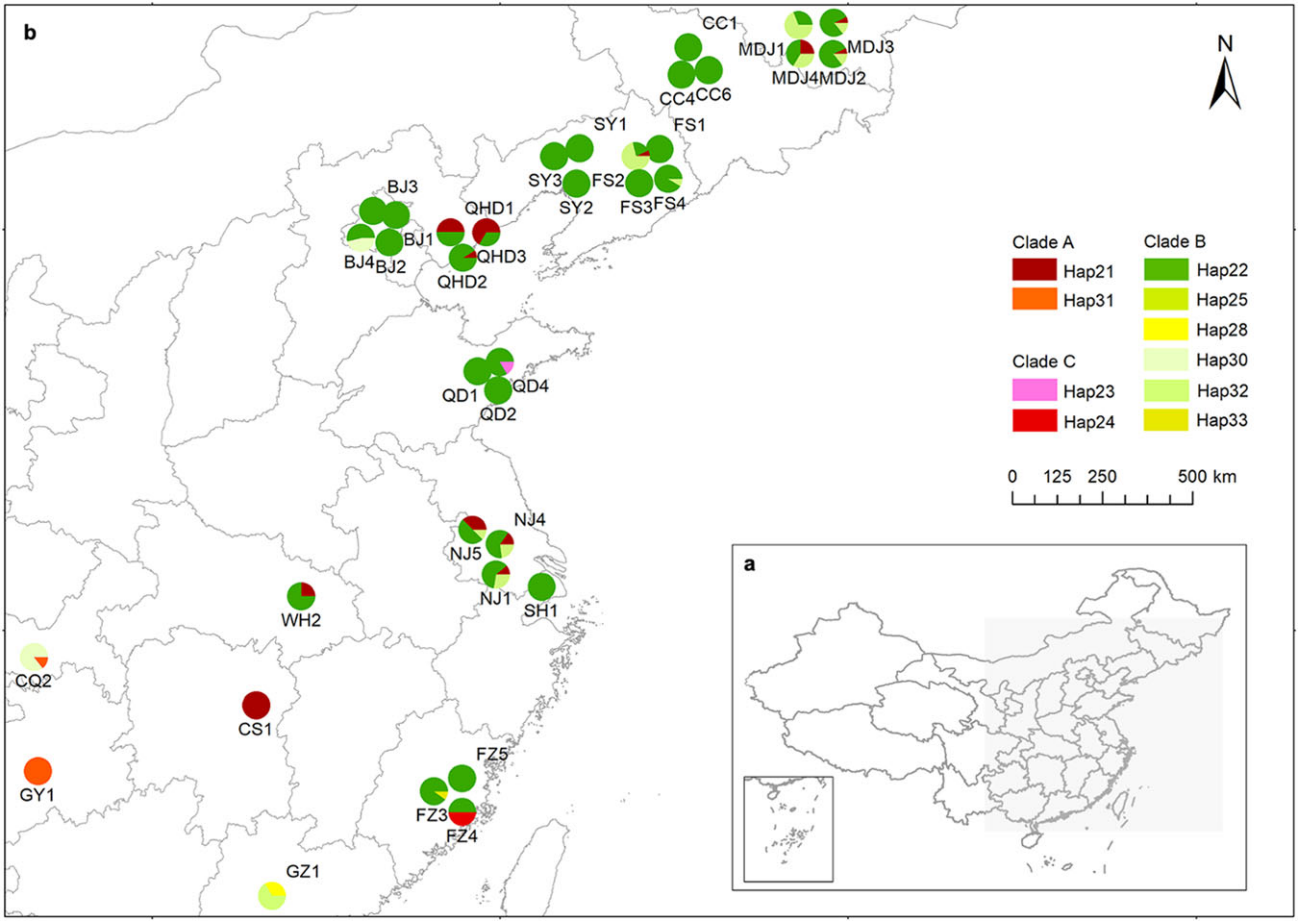
Geographical patterns of cpDNA haplotypes in East China cities. **a** Study area. **b** Genetic patterns, the color proportions of the pie indicated the frequency of each cpDNA haplotype in the population. Abbreviations: Mudanjiang (MDJ), Changchun (CC), Shenyang (SY), Fushun (FS), Qinhuangdao (QHD), Beijing (BJ), Qingdao (QD), Shanghai (SH), Nanjing (NJ), Wuhan (WH), Changsha (CS), Fuzhou (FZ), Guangzhou (GZ), Chongqing (CQ), Guiyang (GY). Numbers are ids of the populations collected in the city.

We identified ten cpDNA haplotypes based on indels and polymorphisms observed within the 836-bp concatenated alignment in chloroplast intergenic spacers. The values of haplotype diversity (H_D_) ranged from 0 to 0.68, and the values of nucleotide diversity (π) ranged from 0 to 0.00122 (Table 1). Common haplotypes (e.g., Hap 22 and Hap 21) and rare haplotypes (e.g., Hap 23, Hap 30, Hap 32, and Hap 33) co-existed within populations in coastal cities such as Qingdao, Guangzhou, and Fuzhou (Fig. 1). Populations in inland cities such as Chongqing and Guiyang only had rare haplotypes like Hap 30 and Hap 31. Three clades could be identified from the rooted neighbor-joining tree and the median-joining network based on the haplotypes (Supplementary Fig. 2).

**Fig. 2:**
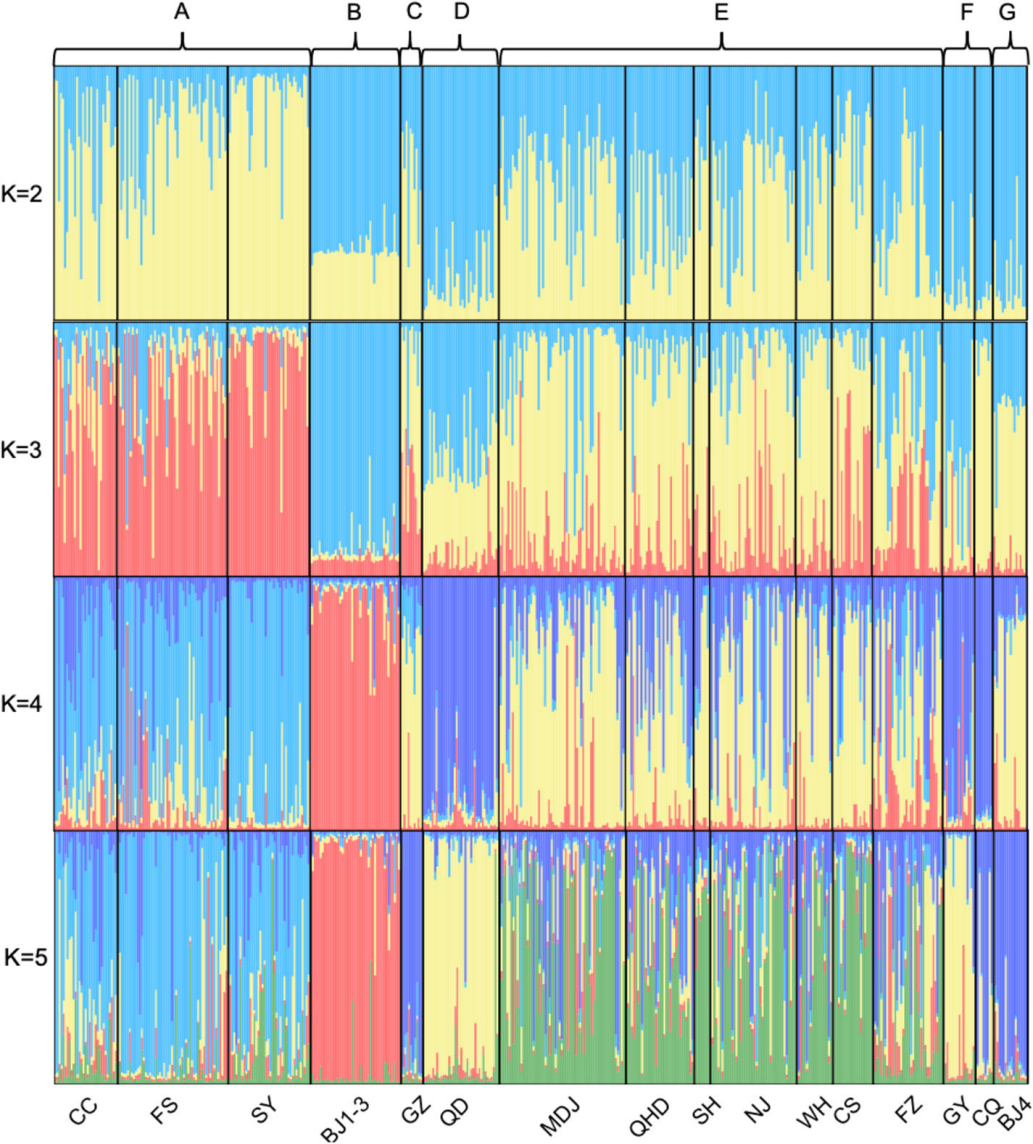
Genetic clusters of the populations of common ragweed in East China cities were inferred using relative probabilities of assignment to genetic clusters in STRUCTURE. Bar heights represent the relative probability of assignment of individual plants to varying numbers of genetic clusters. Genetic clusters identified by GENELAND were indicated by letters. Abbreviations are names of sampled cities and populations: Mudanjiang (MDJ), Changchun (CC), Shenyang (SY), Fushun (FS), Qinhuangdao (QHD), Beijing (BJ), Qingdao (QD), Shanghai (SH), Nanjing (NJ), Wuhan (WH), Changsha (CS), Fuzhou (FZ), Guangzhou (GZ), Chongqing (CQ), Guiyang (GY).

The results from GENELAND and STRUCTURE jointly indicated that common ragweed populations in East China cities could be classified into seven genetic clusters (Fig. 2). The posterior probability density calculated in GENELAND showed mixing around an apparent plateau at the value *k* = 7 (Supplementary Fig. 3). The result from STRUCTURE showed that the highest Delta *K* value (Delta *K* = 31) was obtained at *K* = 5, followed by *K* = 2, 4, and 3 (Supplementary Fig. 4). The membership of each population is shown in Supplementary Table 1. The classification of common ragweed populations into the seven clusters was supported by the moderate within-cluster pairwise F_ST_ values and significant or notable pairwise F_ST_ values among most genetic clusters (Supplementary Fig. 5). The pairwise F_ST_ values between Cluster D and E were moderate. Nevertheless, the two clusters were separable based on the result of STRUCTURE.

**Fig. 3:**
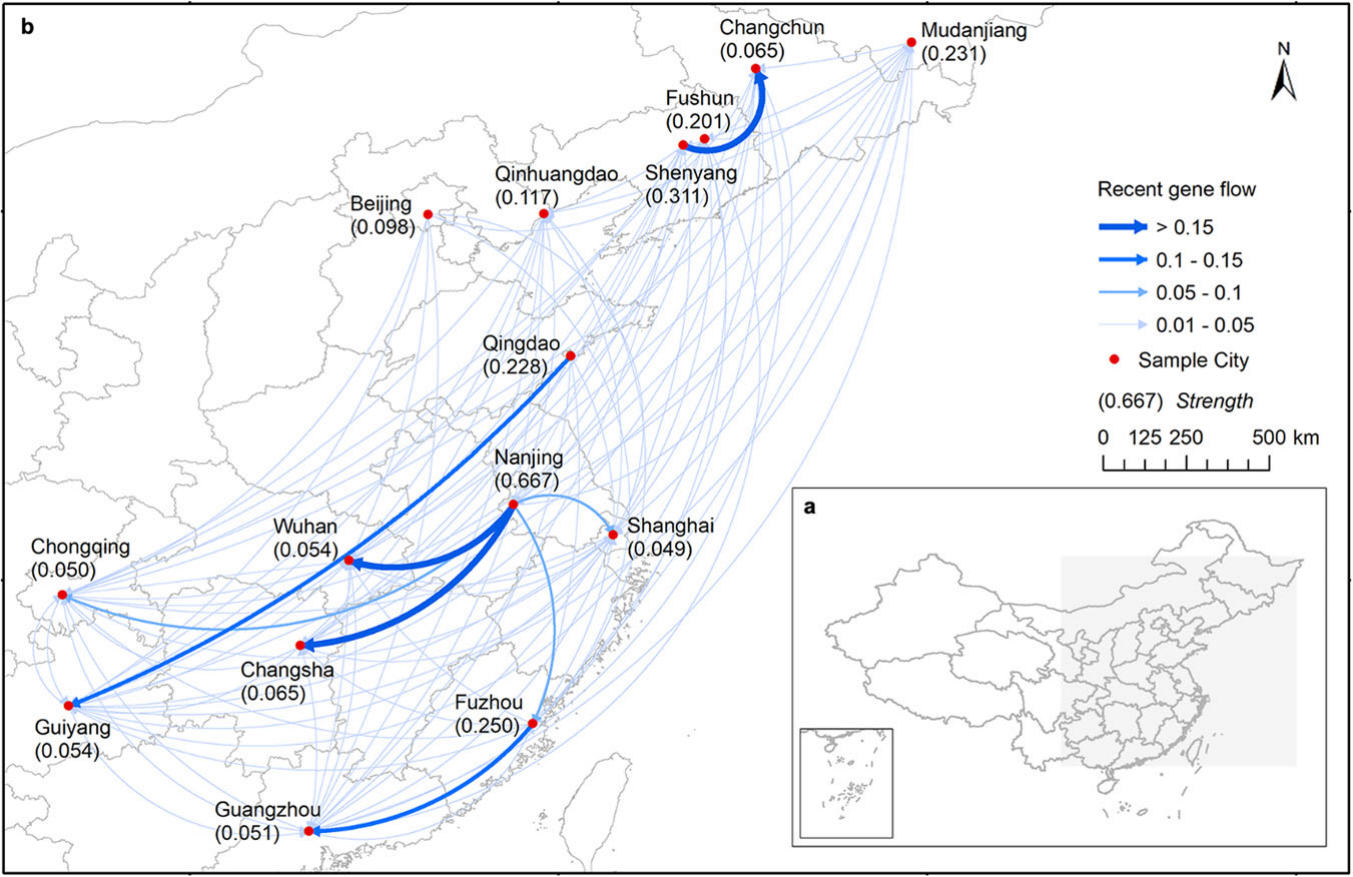
Directed weighted population genetic graph. **a** Study area. **b** Genetic graph, arrow indicates the direction of gene flow, and the width of the edge is weighted by the migration rate. The numbers underneath the city are values of strength, which measure the centrality of the genetic graph.

The migration rates, the fraction of individuals in a population that are migrants derived from another population per generation, indicated that the metapopulations from Nanjing and Shenyang exported more current gene flows than the metapopulations from other cities (Fig. 3). The strength values were also much higher in Nanjing and Shenyang than in the remaining cities. Migration rates between paired cities can be found in Supplementary Table 2.

### The similarity of common ragweed populations in East China cities to those in other countries

The common ragweed populations in East China cities were compared to those in North America and two European countries, France and Italy, where the species is an alien invasive plant (i.e., the clusters in Table 2). According to the result of GENECLASS2, at least 74% of individual plants of the genetic clusters in cities in East China were assigned to the modern North American cluster 2. The remaining individuals were assigned to the modern North American cluster 1 (no more than 21%), and the historical North American cluster (no more than 10%) in each genetic cluster. All individuals in cluster C belong to the modern North American cluster 2 while cluster F has the highest proportion of individuals from historical North American cluster (10%). No individual plant was assigned to the French and Italian clusters.

**Table 2:** Assignment frequencies of individual plants in the seven clusters in East China cities to North American, and French and Italian clusters.

Genetic clusters in other regions	Genetic clusters in East China cities							All
	A	B	C	D	E	F	G	
Modern North American cluster 1	0.205	0.086	0.000	0.120	0.078	0.050	0.077	0.110
Modern North American cluster 2	0.739	0.886	1.000	0.840	0.892	0.850	0.846	0.847
Historical North American cluster 1	0.023	0.029	0.000	0.000	0.012	0.000	0.000	0.014
Historical North American cluster 2	0.034	0.000	0.000	0.040	0.018	0.100	0.077	0.028
French and Italian cluster	0.000	0.000	0.000	0.000	0.000	0.000	0.000	0.000

Modern North American cluster 1 included Connecticut, Maine, Massachusetts, New Hampshire, Pennsylvania, New Jersey, and Rhode Island. Modern North American cluster 2 included Arkansas, Florida, Georgia, Illinois, Iowa, Louisiana, Michigan, Minnesota, Missouri, Ohio, South Carolina, Tennessee, Wisconsin, Quebec, and Nova Scotia. Historical North American cluster 1 included Connecticut, Maine, Massachusetts, New Hampshire, Pennsylvania, New Jersey, Rhode Island, Illinois, Iowa, Minnesota, Ohio, South Carolina, Tennessee, and Wisconsin. Historical North American cluster 2 included Arkansas, Florida, Georgia, Michigan, Ontario, Louisiana, and Missouri^19^.

The expected heterozygosities (H_E_) of the populations in East China cities were not significantly different from those of the modern North American populations (*p* = 0.716, df = 81, *F* = 0.134, Supplementary Fig. 6) and the French and Italian populations (*p* = 0.137, df = 39, *F* = 2.305, Supplementary Fig. 6). The insignificant difference indicated no reduction of genetic diversity in the populations in East China cities. The inbreeding coefficients (F_IS_) of the populations in East China cities were significantly lower than those of the modern North American populations (*p* = 0.027, df = 81, *F* = 5.11; Supplementary Fig. 6). The low F_IS_ value might suggest that the populations in East China cities had lower inbreeding levels than the modern North American populations.

### Patterns inferred from the occurrence records of common ragweed

**Fig. 4:**
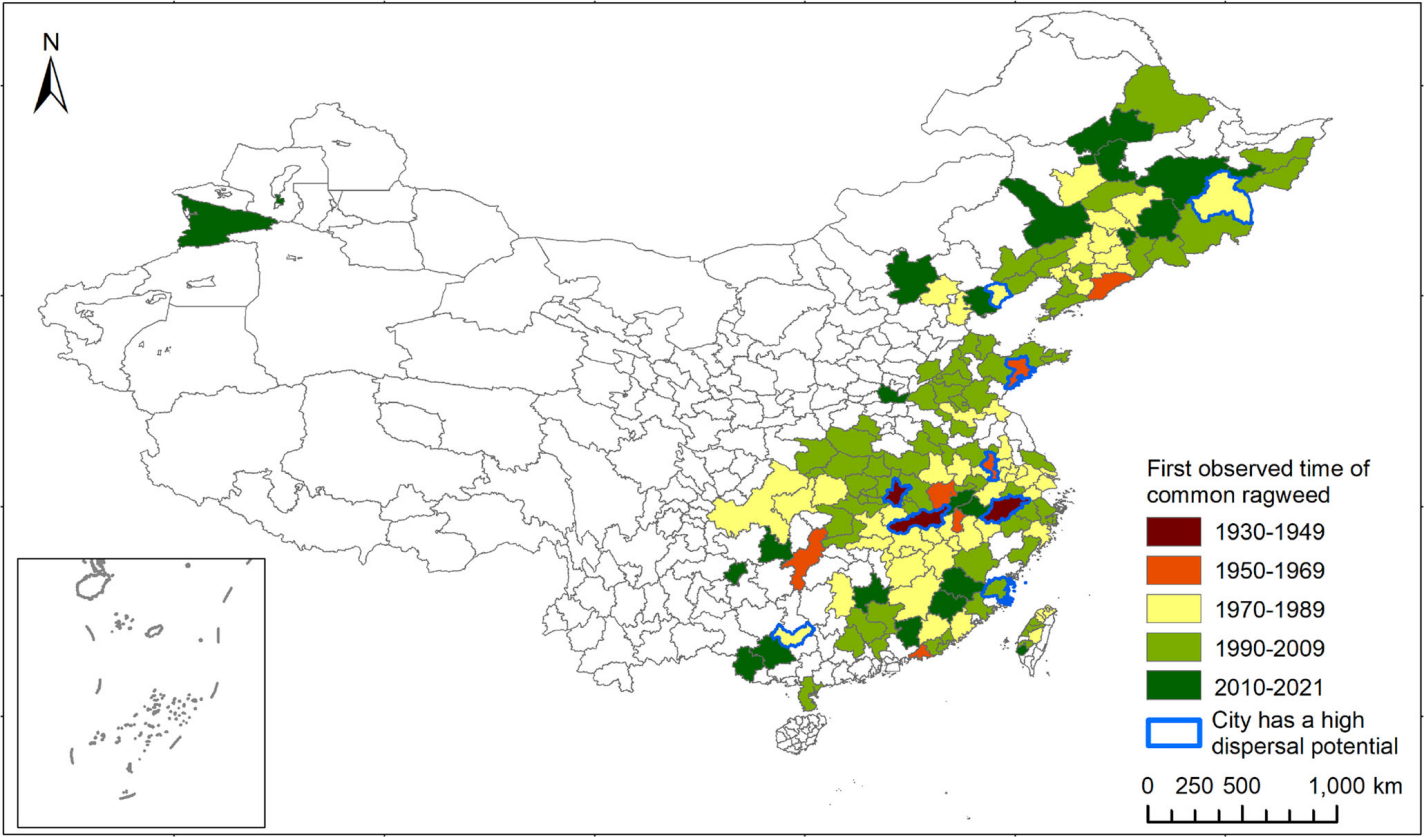
The first observation time (20-year scale) of common ragweed in different regions of China.

About 157 prefecture-level regions in China have reported the occurrences of common ragweed since 1930 (Fig. 4). The occurrence records showed that common ragweed first appeared in the Yangtze River region and then in other parts of China. Records from 1935 to 1949 were mainly along the Yangtze River. From 1970 to 1989, common ragweed appeared in regions further away from the Yangtze River, northeastern China, and the Taiwan Province. Hangzhou (first recorded in 1935), Wuhan (1946), Jiujiang (1948), Nanjing (1958), Qingdao (1964), Mudanjiang (1973), Qinhuangdao (1978), Laibin (1985), and Fuzhou (1991) could be identified as regions that have a high potential for dispersal.

### Grain trade between the United States and China

**Fig. 5:**
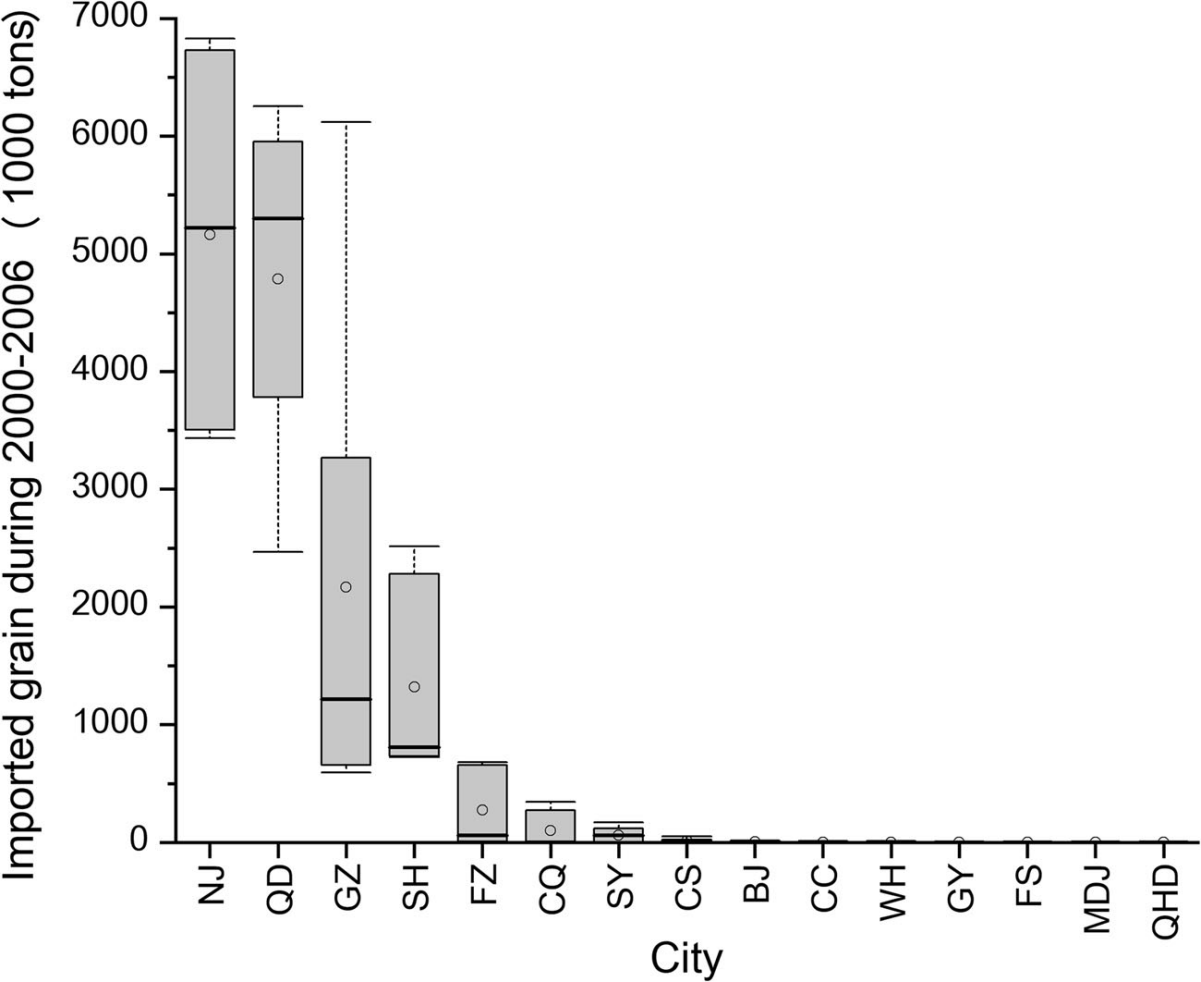
Annual grain imports in East China cities from 2000 to 2006. Horizontal black bars represent the median value. Box limits indicate 75% and 25% quartiles. Whiskers indicated the minimum and maximum value. The white point indicated the mean value. The sample size for each city is 7 (*n* = 7). Abbreviations are names of sampled cities: Mudanjiang (MDJ), Changchun (CC), Shenyang (SY), Fushun (FS), Qinhuangdao (QHD), Beijing (BJ), Qingdao (QD), Shanghai (SH), Nanjing (NJ), Wuhan (WH), Changsha (CS), Fuzhou (FZ), Guangzhou (GZ), Chongqing (CQ), and Guiyang (GY). (Source: EPS CHINA DATA).

From 1999 to 2009, the trade data showed that the states in the modern North American cluster 2 exported more grain products to China than those in the modern North American cluster 1 (Table 3). Importation of grains in the 15 cities showed that Nanjing and Qingdao received the most grain products from 2000 to 2006 (Fig. 5). From 1935 to 1939, the trade data revealed that Austrila, French indo-China and Thailand were the primary source countries for grain product imports to China (Supplementary Table 3).

**Table 3:** Export of grain products from states included in the two modern North American genetic clusters to China from 1999 to 2009 after being adjusted by the Consumer Price Index (CPI, Year 2000 = 100) (Source: Foreign Trade Division, U.S. Census Bureau).

Year	Modern North American cluster 2		Modern North American cluster 1	
	Value (million dollars)	Percentage of total value (%)	Value (million dollars)	Percentage of total value (%)
1999	340.45	99.96	0.14	0.04
2000	820.34	99.89	0.90	0.11
2001	523.63	99.92	0.43	0.08
2002	638.35	99.95	0.30	0.05
2003	1882.65	99.99	0.21	0.01
2004	1971.19	99.98	0.48	0.02
2005	1732.08	99.93	1.28	0.07
2006	2026.50	99.99	0.19	0.01
2007	1526.42	99.98	0.34	0.02
2008	1767.33	97.41	46.95	2.59
2009	3168.65	94.40	187.84	5.60

## Discussion

The assignment tests suggested that the modern North American cluster 2 was likely the primary source of common ragweed populations in East China cities. The states within the distribution range of the modern North American cluster 2 exported far more grain products to China than other states. Studies showed that grain products in the U.S. retained many common ragweed seeds^29,30^. More than 90% of the grains imported by China are for consumption^31^. They were processed into food, feed, or alcohol in factories in urban areas^32^. Thus, grains contaminated by common ragweed seeds were primarily transported into urban areas. During transportation, grains can leak from vehicles^33,34^. The airflow created by vehicles can also facilitate the dispersal of common ragweed-contaminated grains^35–37^. Those processes can allow common ragweed seeds to escape into urban environments. Consequently, the propagule pressure of common ragweed in urban areas is high, contributing to the successful colonization of urban areas. Only a small portion of the imported grain in China is used as planting seeds^31^. These seeds often undergo careful screening before cultivation to ensure the desired species are planted, with non-target plants being removed during the growing process^38^. As a result, it is unlikely that seeds for planting are the primary source of common ragweed propagules in cities in East China. The impact of grain trade on the introduction source of common ragweed is also reflected in genetic clusters. For example, all individuals in cluster C belong to the modern North American cluster 2. This may be because all individual plants in cluster C were collected from Guangzhou, which is a major port for grain import. Our results corroborate the conclusion that introduction through contaminated food commodities is one of the presumed routes of IAS invasion^39^.

The assignment test results suggested that the historical North American clusters (collected during 1873 to 1939) were a minor source of common ragweed populations in cities in East China. Common ragweed was first recorded in China in 1935^22^. Historical grain trade facilitated the introduction. Mainland China mainly imported grains from Australia, French Indo-China (including Vietnam, Cambodia, and Laos), Thailand, Kwangchowwan leased territory (a territory of China at the southern tip of the Liaodong Peninsula of Manchuria, leased to Japan in 1905), the U.S., Myanmar, Korea, Canada, and Argentina during 1935-1939^40,41^ (Supplementary Table 3). The occurrences of common ragweed were first recorded in Vietnam, Cambodia, Laos, Thailand, Kwangchowwan leased territory, Myanmar, and Korea later than 1939^1,42^. Therefore, those countries and regions are less likely the initial source. Common ragweed is native to the U.S., and Canada, and was introduced to Argentina before 1820^43^. Also, the species was first recorded in Japan in 1877 and established in the country in the 1930s^44^, while it was first recorded in Australia in 1908^45^. Common ragweed could be directly introduced into China from the U.S., Canada, and Argentina or indirectly from Japan and Australia. Since genetic data on common ragweed in Argentina, Japan, and Australia are unavailable, it is difficult to validate those possibilities. Nevertheless, the bridgehead introduction passage for common ragweed between Europe and Australia have already been identified^18^, our findings point to possible bridgehead introduction passages between China and other countries.

The minor role of the historical North American clusters as invasion sources could be attributed to the repeated modern introductions in East China cities and genetic exchange among cities. These findings support the possibility that modern genotypes have gradually replaced the genotypes introduced through historical introductions. Cluster F has the highest proportion of individual plants from the historical North American cluster. Individuals in cluster F came from Guiyang and Chongqing, which are in the inland region of China. The spread of modern genotypes of common ragweed to inland areas takes a longer time, resulting in a greater retention of historical genotypes. A previous study found that the proportion of historical North American clusters in the sources of non-urban common ragweed populations in China was three times that of modern North American clusters^21^. This can be attributed to the less developed transportation networks in non-urban areas, where populations of non-urban common ragweed interact less with populations from other areas. Thus, more historical genotypes retained. We offered an potential explanation to the early findings.

These findings support the hypothesis that international trade introduced common ragweed into urban areas in East China cities. We inferred that modern grain exportation from the United States to China dominated the introduction process. While economic variables such as grain importation are not directly related to the establishment of IAS, they integrate the effect of variables that directly affect the outcome of invasion, such as propagule pressure and pathways of introduction^46,47^. Although other studies have identified grain trade as an introduction pathway of IAS to cities through occurrence records^48^, our results provide new evidence from a population genetic perspective.

The existence of both the historical and modern North American clusters as invasion sources pointed to multiple introductions of common ragweed in East China cities that occurred in different periods. The spatial genetic pattern of common ragweed populations in East China cities showed that this possibility is highly likely. High genetic variability, low mean F_IS_, and rare bottleneck effects were observed in the populations in East China cities. Multiple introductions often lead to high genetic variability in invasive species^17^. The relatively low mean inbreeding coefficient (F_IS_ = 0.342) suggests significant genetic differentiation among parental plants in most populations, which could be attributed to multiple introductions^49^. Rare bottleneck effects indicate that most populations did not experience a substantial reduction in population size. For species at the introduced range, the recovery of their gene pool may be replenished through multiple introductions^15^. These observations support the occurrence of multiple introductions.

Furthermore, we observed that populations from distant cities (e.g., Mudanjiang, Qinhuangdao, and Wuhan) were grouped together in the same genetic cluster. However, contemporary gene flows among these populations were low. The limited gene exchange between distant cities suggests that their assignment to the same genetic grouping is more likely a result of high genetic similarity among invasive source populations rather than recent gene flows^50^. In other words, the similar invasive source was introduced multiple times into those cities.

We also found that introductions occurred more frequently in coastal areas than inland. The co-existence of common and rare haplotypes from different clades in coastal cities indicated possible multiple introductions. The sole existence of the rare haplotype in inland cities suggested less frequent introductions. According to the literature, most areas of East China are considered suitable habitats for common ragweed^23,51,52^. Higher international trade activities in coastal areas than inland areas might explain this pattern because the trade increases the propagule pressure. Previous studies also found that common ragweed populations in Eastern and Western Europe were related to different regions of North America due to differences in trade patterns^16,17^.

Occurrence records also indicated a high likelihood of multiple introductions. For example, Qingdao first recorded common ragweed in 1964, and Fuzhou recorded the first observation in 1991^53,54^. The two cities are far from each other (1118 km), making natural dispersal less likely. Also, the result of the genetic analysis showed that common ragweed populations from Qingdao and Fuzhou belonged to different genetic clusters. These findings support the possibility that common ragweed invaded East China cities through multiple introductions at different times.

In the spreading stage, the dispersal of common ragweed between cities in East China primarily might depend on stowaways associated with anthropogenic transportation. Studies have demonstrated that road networks and vehicle airflows promote the spread of common ragweed seeds^36,55^. In this study, we observed high levels of gene flow from Shenyang to Changchun (0.1544), Qingdao to Guiyang (0.1149), and Fuzhou to Guangzhou (0.1461). Those cities are connected by dense road networks.

Furthermore, the movement of soil and mowing machines may contribute to seed spread^1^. Common ragweed seeds can remain viable in the soil for up to 40 years^45^, and a single common ragweed plant can produce up to 6000 seeds^56^. Once established in an area, common ragweed can produce a large number of seeds that contaminate the soil over an extended period. If the invaded area is a center of horticulture or agriculture, common ragweed seeds can inadvertently be transported to other areas through the movement of soil and mowing machines. Among the studied cities in East China, Nanjing is one of the most significant horticulture centers in China, which may contribute to the high gene flow observed between Nanjing and other cities, e.g., Changsha (0.1721), Wuhan (0.1651), Shanghai (0.0889), Fuzhou (0.06), and Chongqing (0.0518).

Lastly, natural dispersal may have played a role in the spread of common ragweed seeds between cities. Barochory is the primary way of natural dispersal of common ragweed seeds^1^. However, the barochory distance of common ragweed seeds in terrestrial habitats was found to be less than 1 meter per year^36^. Hydrochory has been identified as a secondary mode of natural seed dispersal for common ragweed within its native range^10,56^. Its role in the dispersal of common ragweed seeds among East China cities worth further studies. We detected significant gene flow from Nanjing to Changsha (0.1721) and from Nanjing to Wuhan (0.1651). Those cities are connected by waterways. However, for other cities with significant gene flows, no direct waterway connections exist among them.

Some studies suggest that pollen may play a role in gene exchange among cities. For instance, research conducted in Europe has demonstrated that common ragweed pollen can be dispersed over several hundreds or even thousands of kilometers through the air^57–59^. However, it is important to note that the pollination rate of common ragweed pollen originating from distant locations thousands of kilometers away would be extremely low, given that common ragweed populations were not widely distributed in the cities examined in our study.

Shanghai, Guangzhou, Qingdao, and Fuzhou emerge as potential main sites of introduction. These cities, being port cities, have a substantial grain importation volume. Previous studies have suggested that port cities are more likely to serve as entry points for the introduction of alien species^60^. The common ragweed populations in these cities exhibit a mixture of common and rare cpDNA haplotypes from different clades, and their inbreeding coefficients are low. However, as a result of effective control measures implemented in recent years^2^, the genetic diversity of Shanghai’s population is low. In contrast, the populations in Qingdao, Guangzhou, and Fuzhou maintain high levels of genetic diversity. Consequently, the potential risk posed by these latter populations are therefore greater.

Nanjing and Shenyang are identified as potential primary centers for the spread of common ragweed. Our findings demonstrate significant gene flows originating from the metapopulations of common ragweed in these cities to other cities in East China. This can be attributed to the prominent roles of Nanjing and Shenyang in grain importation. Nanjing ranks first in imported grain volume among all the studied cities, while Shenyang surpasses other cities within the same genetic cluster in terms of grain imports. Shenyang’s proximity to Yingkou, a historically crucial grain importation port^61,62^, further strengthens its influence. In addition, both Nanjing and Shenyang serve as key transportation hubs in China^63,64^, contributing to their dominance in gene flows along the transportation network. Notably, Nanjing’s floriculture adds another pathway for seed dispersal^65^, as soil containing common ragweed seeds can be inadvertently transported to major cities through floriculture products.

Previous studies listed Wuhan, Nanjing, and Shenyang as cities with a high spread potential of common ragweed^24^. The gene flow pattern did not support the designation of Wuhan as a spread center. Wuhan’s spread potential may have been significantly reduced due to effective biocontrol programs applied in recent years^24,66^. Another possibility is the small sample size in Wuhan because only one urban population was located in Wuhan. In addition to the cities mentioned above, we inferred that Hangzhou, Jiujiang, Qingdao, Mudanjiang, Qinhuangdao, Laibin, and Fuzhou had high spread potentials of common ragweed in China based on the temporal information of occurrence data. Among these cities, samples collected from Fuzhou, Qingdao, Mudanjiang, and Qinhuangdao were analyzed, but the metapopulations in Mudanjiang, and Qinhuangdao had low gene flows to nearby sampling cities in East China. The results of the genetic analysis did not corroborate with the inferences based on the occurrence records. The discrepancy might be due to the subjectivity of the inference method and the uncertainties associated with historical occurrence data.

Findings from this study can contribute to the control of common ragweed in the following aspects: (1) Our results show that urban areas can serve as landing points for and spread hubs of common ragweed. In order to control common ragweed, controlling the spread among urban areas should not be left out of the overall control program. (2) There is plenty of room for improvement in quarantine work at Chinese port cities because the primary source of common ragweed might be from the modern North American clusters. Also, coastal cities have experienced more introduction events than inland cities. Strict inspection and quarantine of grains imported from regions that are known sources of common ragweed seeds should be practiced in ports that handle grain importation. Monitoring programs and awareness campaigns should be set up in these cities to monitor common ragweed populations. Waste from factories that process imported grains must be treated before final disposal.

While our study contributes a new understanding of the invasion pathway of common ragweed in urban areas, several limitations should be addressed in future studies. First, although our field campaign covered the East China cities, more samples from more cities can further reduce uncertainty in the results. Second, we could only obtain genetic data for North America and two European countries after searching through the major international genetic databases and contacting authors who published papers on common ragweed’s genetics. Due to the lack of data, we could not explore the contribution of other potential sources to the genetic pattern of common ragweed populations in East China cities. For example, we could not confirm the bridgehead introduction passage from Argentina, Japan and Australia due to the lack of genetic data on common ragweed in these countries.

Knowledge of the invasion pathway of common ragweed in urban areas is crucial for controlling this IAS. However, limited information is available because studies have not adequately addressed this topic. This study examined the spatial genetic pattern of common ragweed plants in urban areas in East China cities. We also explored the possible role played by the grain trade between China and the U.S. in shaping the genetic pattern. The results indicated that common ragweed in East China cities had experienced multiple introductions at different times. We inferred that the modern-day grain trade between the U.S. and China could be the primary source of propagule. Finally, we identified cities that might serve as spread centers by examining the gene flows among the cities and the temporal information in occurrence records. Our findings contribute new knowledge on the invasion pathway of common ragweed in urban areas. Also, the information gained in this study is useful for controlling common ragweed in urban areas in China and other countries. Nevertheless, the entire study should be viewed only as the beginning of efforts to reveal invasion pathways of common ragweed and other IAS in urban areas. More studies combining the strength of observations with population genetic methods are needed to understand the pathways and driving mechanisms better.

## Methods

### Study area

The study area includes cities in East China (Supplementary Fig. 7). East China holds most of the Chinese population^25^ and is the main suitable habitat for common ragweed^51^. To locate the current distribution of common ragweed populations in cities, we referred to the literature^15,67,68^ and consulted local experts. Also, we checked the geographic locations of occurrence records of common ragweed contained in the Chinese Virtual Herbarium (CVH)^69^. Based on the compiled occurrence records, we selected 19 East China cities with reliable records of common ragweed to conduct field surveys and were able to find urban populations of common ragweed in 15 cities.

### Data collection

We collected samples from 37 common ragweed populations in the 15 cities between 2017 and 2018 (Supplementary Table 1). The sampled populations were located in urban areas and were at least 3 km from each other. According to the varied population size of urban populations, 6–19 plants were sampled at least 2 m from each other inside each population. We collected at least five fresh leaves from each sampled plant and stored the leaves in sealed plastic bags filled with silica beads for desiccation. We recorded the longitudes and latitudes of sampling sites using a handheld GPS unit (AGM X2, with an accuracy of 10 m) during the field campaign.

Genomic DNA was extracted from the leaf samples using the CTAB method^70,71^. We also obtained the genomic DNA of four French and Italian populations and one North American population from Ciappetta et al.^72^. Six microsatellite markers were used to mark the genomic DNA of the common ragweed populations mentioned above. To ensure compatibility among Chinese, French and Italian, and North American populations, the microsatellite markers used in this research were the same as those used by Martin et al.^19^. Microsatellite primer sequences were Gen-Bank sequences FJ595149 (Ambart04), FJ5950 (Ambart06), FJ5952 (Ambart17), FJ5953 (Ambart18), FJ595155 (Ambart24), and FJ595156 (Ambart27) (Supplementary Table 4). In-house ROX-labeled size standards were used for genotyping^73^. GENEMarker version 1.5 was utilized for microsatellite loci fragment analysis and allele calling. Microsatellite loci fragment data of modern and historical North American populations collected from Martin et al.^19^ were re-read for data consistency^19^.

We sequenced chloroplast DNA (cpDNA) of the 37 East China populations. The two spacers (atpH–atpF and psbK–psbI) of chloroplast DNA spacer regions in common ragweed were chosen for analysis (Supplementary Table 5). Chloroplast DNA chromatograms of sequenced samples were aligned using MAFFT^74^ and Geneious version 11.0.4 (Biomatters Ltd., Auckland, New Zealand).

To further trace the invasion sources of common ragweed populations in East China cities, we obtained microsatellite loci fragment data of 45 North American populations and 426 historical herbarium individual plants (Supplementary Table 6) from Martin et al.^19^. The data was collected in the summer of 2009.

DNA extraction, amplification, and sequencing of nuclear microsatellites and cpDNA were completed by the Bio-ulab company (Beijing, China) (Details of the procedure were included in the supplementary materials).

### Data analysis

Tests of genotypic linkage equilibrium and estimation of null allele frequency were performed in GENEPOP 4.7.0^75^. Genetic diversity indices, including the number of different alleles (N_A_), expected heterozygosity (H_E_), and inbreeding coefficients (F_IS_) were estimated in GenAlEx 6.51 and GENEPOP 4.7.0^75,76^ to represent diversity at nuclear microsatellite loci, respectively. Deviations from Hardy-Weinberg equilibrium of sampled populations were assessed using F_IS_^77^, and their significance was tested using GENEPOP 4.7.0^75^. The potential bottleneck of sampled populations was detected using BOTTLENECK version 1.2.0.2^78^.

We aligned the atpH–atpF and psbK–psbI sequences of common ragweed from CBOL Plant Working Group^79^ with our haplotypes to determine the correctness of our sequence. The number of haplotypes (*h)*, haplotype diversity (H_D_), and nucleotide diversity (π) of sampled populations were computed using DnaSP 6^80^. Except for software packages specifically noted, all statistical analyses were carried out using the basic R 4.0.2^81^.

The Bayesian clustering algorithms contained in STRUCTURE version 2.3.3^82^ and GENELAND version 4.0.3^83^ were used to cluster common ragweed populations based on the nuclear microsatellite and the cpDNA haplotype (details of the parameters were in the supplementary materials). Pairwise population-specific fixation indices (F_ST_) between sampled populations were calculated based on nuclear microsatellite markers. Pairwise population F_ST_ were calculated in the diveRsity package^84^.

The geographical distribution of cpDNA haplotypes was visualized using ArcGIS Desktop 10.2 (ESRI, Redlands, CA, USA). A rooted neighbor-joining tree with *Ambrosia trifida* L. as an outgroup was constructed using Geneious version 11.0.4 (Biomatters Ltd., Auckland, New Zealand) with a Tamura-Nei genetic distance model using 1000 replicates. A median-joining network of haplotypes was generated by using NETWORK 10.1^85^. The tree and the network were used to explore the relationships among cpDNA haplotypes.

The gene flows among the sampled cities were estimated using BayesAss v3.0.4^86^. A genetic graph was built using the gene flows among the sampled cities. The R packages POPGRAPH^87^ and IGRAPH^88^ were used to construct the graph and calculate graph centrality measures. More details of the analyses can be found in the supplementary materials.

Genetic diversity indices, including H_E_ and F_IS_ of French and Italian, and North American populations, were estimated in GenAlEx 6.51 and GENEPOP 4.7.0^75,76^, respectively. Analysis of variance (ANOVA) was used to test differences among the geographic ranges in H_E_ and F_IS_ using the *stats* package^81^. In the previous study, the source populations of common ragweed in East China was identified^21^. This study further investigated the primary source of propagule and dispersal centers for common ragweed in cities in East China. Each plant was assigned to French and Italian, modern North American, and historical North American populations. The North American samples were grouped by the genetic clusters identified by Martin et al.^19^. The historical clusters are comprised of herbarium specimens collected between 1873 and 1939, while the modern clusters are consisted of samples collected in 2009^19^. The assignment was performed in the GENECLASS2 software^89^.

Because this study focused on exploring the role of the grain trade in the introduction of common ragweed to East China cities, we collected exportation and importation data. Exports of grain products from states in the United States to China from 1999 to 2009 were provided by the Foreign Trade Division, the U.S. Census Bureau. The exports were adjusted by using the Consumer Price Index. This time range was chosen because the U.S. Census Bureau has provided data on grain trade to China since 1999, and the genetic analysis of the invasion source was based on modern North American common ragweed genetic data collected in 2009^19^. Data collection and analysis were conducted using Excel.

Data on grain importation from 2000 to 2006 in studied cities were collected from EPS CHINA DATA (http://microdata.sozdata.com/home.html). The time range was chosen because China’s customs data on grain imports were available in EPS CHINA DATA from 2000, and China’s customs began to implement stricter quarantine measures for common ragweed seed in 2007. Data on China’s imports of grain products from 1935 to 1939 were collected from *Chinese Maritime Customs Historical Material: 1859-1948*^39^. This time range was selected due to the historical North American genetic data being based on herbarium specimens collected between 1873 and 1939. In addition, common ragweed was first recorded in China in 1935^22^, and importation data prior to 1935 utilized a different statistical scope compared to the later years. Data collection and analysis were performed using the *stringi* and the *dplyr* package^90,91^.

We searched occurrence data of common ragweed from databases such as the Global Biodiversity Information Facility (GBIF)^92^, CVH^42^, Plant Photo Bank of China^93^, and the Plant Specimen Database^94^ until June 07. 2021. The data was supplemented with survey data, anecdotal descriptions, and images from literature. We removed occurrence records in rural areas. We then generated the temporal and spatial distribution map of common ragweed in China using ArcGIS Desktop 10.2. We defined the first present time of common ragweed occurrence data in the prefecture-level city as the first observation time of common ragweed in that prefecture-level city. We inferred a city as a regional first landing point when common ragweed plants were observed in the city earlier than any other adjacent city. Bullock et al.^10^. assigned areas with more distribution records of common ragweed as the spread center because they had higher dispersal potential. Marchioro et al.^95^. assumed that if areas adjacent to an invasion area is suitable for the invasive species, the invasion area is designated as the spread center. Building upon the methods used in above studies, we classified a city as the potential spread center if it met three criteria: (1) The city first reported the occurrence of common ragweed in the region; (2) It has consistent occurrence records over a 20-year period; (3) Its adjacent cities are suitable habitats for common ragweed.

### Statistics and reproducibility

Genetic diversity indices (N_A_, H_E_, F_IS_, *h*, H_D_ and π) were estimated in GenAlEx 6.51^76^, GENEPOP 4.7.0^75^ and DnaSP 6^80^. The potential bottleneck was detected using BOTTLENECK version 1.2.0.2^78^. STRUCTURE version 2.3.3^82^ and GENELAND version 4.0.3^83^ were used to cluster common ragweed populations. The rooted neighbor-joining tree was constructed using Geneious version 11.0.4. The median-joining network of haplotypes was generated by using NETWORK 10.1^85^. The gene flows were estimated using BayesAss v3.0.4^86^. The assignment was performed in the GENECLASS2 software^89^. Except for software packages specifically noted, all statistical analyses were carried out using the basic R 4.0.2^81^. The specific details of the analyses and sample sizes are described in the methods above.

### Reporting summary

Further information on research design is available in the Nature Portfolio Reporting Summary linked to this article.

### Supplementary information

Supplementary information

Reporting Summary

## Data Availability

Microsatellite genotypes, chloroplast haplotypes, input files used in the analysis, and the source data behind the graphs in Figs. 1,2,4,5 are accessible through the Mendeley Data with identifier DOI:10.17632/kxgtk8r5kz.1.Chloroplast intergenic spacer locus sequences that support the findings of this study have been deposited in GenBank with the accession numbers MZ826750–MZ826763.The source data behind the graphs in Fig.3 are available in Supplementary Table 2.

## Code availability

R codes used in the analysis are accessible through the Mendeley Data with identifier 10.17632/kxgtk8r5kz.1.
