# Supplementary material for: China-US grain trade shapes the spatial genetic pattern of common ragweed in East China cities
Source: Commun Biol. 2023 Oct 21;6:1072. doi: 10.1038/s42003-023-05434-5 (PMC10590438; doi:10.1038/s42003-023-05434-5)
Supplement: Supplementary file 1 — Supplementary information [file 42003_2023_5434_MOESM1_ESM.pdf]

Supplemental Information for:

China-US grain trade shapes the spatial genetic pattern of common ragweed in East China cities

This PDF file includes:

Supplementary Text
Figs. S1 to S7
Tables S1 to S6

Supplementary Text

Methods

DNA extraction, nuclear microsatellite and cpDNA amplifications, and fragment sizing

The CTAB method was used to extract genomic DNA from silica-dried leaf tissue from 37 populations in East China cities (Lee, Milgroom, & Taylor, 1988; Zhang, Zhang, Liu, Wen, & Wang, 2010). In a brief, the cell walls of leaves were broken down in the presence of liquid nitrogen. The CTAB extraction buffer was then added, and after incubation at 65 °C, purification with phenol:chloroform (1: 1), precipitation with isopropanol and rinsed with 70% ethanol were conducted. Finally, the DNA was dissolved in 40μl 1 XTE Solution (Sterilized).

Genomic DNA of 37 populations in East China cities, four French and Italian populations, and one North American population were used to amplify and fragment sizing nuclear microsatellites. Microsatellite primer sequences were Gen-Bank sequences FJ595149 (Ambart04), FJ5950 (Ambart06), FJ5952 (Ambart17), FJ5953 (Ambart18), FJ595155 (Ambart24) and FJ595156 (Ambart27) (Supplementary Table 4), in-house ROX-labeled were used as size standards (DeWoody et al., 2004) and implemented Biosystems Hi-DyeR formamide. The PCR profile was as follows: initial denaturation for 5 min at 94 °C; there were 35 cycles of 30 s denaturation at 94 °C; renaturation at 54 ℃ for 35 s; and extension at 72 ℃ for 40 s. Finally, it was extended at 72 ℃ for 3 min.

Genomic DNAs of 37 populations in East China cities were used to sequence two spacer regions (atpH–atpF and psbK–psbI) of chloroplast DNA (cpDNA). The PCR profile was as follows: initial denaturation for 5 min at 94 °C; 35 cycles of 30 s denaturation at 94 °C; renaturation at 55 ℃ for 35 s; and extension at 72 ℃ for 40 s. Finally, it was extended at 72 ℃ for 3 min. In total, we have 467 chloroplast DNA sequence chromatograms. Due to high AT content and poly A structure, 29 sequence chromatograms were excluded from analysis. 438 sequence chromatograms were readable and used for alignment. Chloroplast DNA chromatograms of sequenced samples from populations in East China cities were aligned using MAFFT (Katoh & Toh, 2010), then the alignment was manually checked and edited using Geneious version 11.0.4 (Biomatters Ltd., Auckland, New Zealand). In the alignment, the 5' UTR and 3' UTR contain missing and ambiguous sites, so both regions were excluded in the following analyses. All sequences were concatenated and aligned into a super-alignment in Geneious version 11.0.4. (Biomatters Ltd., Auckland, New Zealand).

Genomic DNA extraction, amplification, and sequencing of nuclear microsatellites and cpDNA were completed at Bio-ulab company (Beijing, China).

Genetic pattern of common ragweed in East China cities

Tests of genotypic linkage equilibrium were performed with 10,000 Markov chains, 100 batches, and 50,000 iterations per batch implemented in GENEPOP 4.7.0 (Rousset, 2008) for each pair of loci. The EM algorithm implemented in GENEPOP 4.7.0 (Rousset, 2008) estimated the frequency of the null allele. Genetic diversity indices, including the number of different alleles

(N_A), expected heterozygosity (H_E), and inbreeding coefficients (F_{IS}), were estimated in GenAlEx 6.51 and GENEPOP 4.7.0 (Peakall & Smouse, 2012; Rousset, 2008) to represent diversity at nuclear microsatellite loci, respectively. Deviations from Hardy-Weinberg equilibrium were assessed across loci and sampling localities by F_{IS} (Weir & Cockerham, 1984), and significance was tested by 10000 Markov chains, 20 batches, and 5000 iterations per batch implemented in GENEPOP 4.7.0 (Rousset, 2008). The significance of the p-value was assessed by the Bonferroni correction implemented in the basic R version 4.0.2 (R Core Team, 2018). The potential bottleneck was detected by BOTTLENECK version 1.2.0.2 (J. M. Cornuet & Luikart, 1996). The Two-phased Mutation Model (TPM) with variance of the geometric distribution for TPM = 0.36 and proportion of SMM in the TPM = 0.000 was used in BOTTLENECK version 1.2.0.2 (J. M. Cornuet & Luikart, 1996), for it was considered to be the most suitable model for microsatellite data (J.-M. Cornuet & Luikart, 2018). The mode-shift indicator of TPM is used to evaluate the population's bottleneck status. Pairwise population F_{ST} with a 95 % confidence interval were calculated in the diveRsity package (Keenan, McGinnity, Cross, Crozier, & Prodöhl, 2013) with 1,000 permutations.

The number of haplotypes (h), haplotype diversity (H_D), and nucleotide diversity (π) were computed with DnaSP 6 (Rozas et al., 2017). The geographical distribution of cpDNA haplotype was visualized using ArcGIS Desktop 10.2 (ESRI, Redlands, CA, USA) to explore the relation of the populations in East China cities. A median-joining network of haplotypes was generated by NETWORK 10.1 (Bandelt, Forster, & Röhl, 1999) without outgroup with MP options (Bandelt et al., 1999). A rooted neighbor-joining tree with *Ambrosia trifida* L. as an outgroup was constructed using Geneious version 11.0.4 (Biomatters Ltd., Auckland, New Zealand) with a Tamura-Nei genetic distance model using 1000 replicates.

STRUCTURE version 2.3.3 (Pritchard, Stephens, & Donnelly, 2000) and GENELAND version 4.0.3 (Guillot, Estoup, Mortier, & Cosson, 2005) with the Bayesian clustering algorithms were used to implement genetic clustering analyses of the populations in East China cities. They classified individuals into an a priori unknown number of genetic groups using a probabilistic approach based on their multilocus genotypes at the six nuclear microsatellites and the cpDNA haplotypes. STRUCTURE only used prior information about sampling populations, while GENELAND added the longitude and latitude of the sampled populations and implemented spatially explicit algorithms to optimize inference.

The spatial model assuming uncorrelated allele frequencies was implemented in GENELAND version 4.0.3 (Guillot et al., 2005). The maximum number of nuclei in the Poisson–Voronoi tessellation was set to 500. The inference of population structuring was based on 10 independent runs, each allowing the number of clusters to vary between 1 and 10. Each run consisted of 500 000 Markov chain Monte Carlo (MCMC) iterations with a thinning interval of 100. The data were post-processed with a burn-in of 1000 iterations and 500 by 700 cells in the spatial domain. The most likely number of clusters K was chosen by GENELAND according to the density of the posterior probability chain.

The analyses were carried out in STRUCTURE version 2.3.3 (Pritchard et al., 2000) using the admixture model, which assumed correlated allele frequencies but no location priors. Twenty independent runs were performed with 200,000 initial steps of burn-in followed by 1,000,000 iterations for each of K = 1 through 10. The most likely number of clusters K was chosen because it corresponded to the peak of Delta K according to the method of Evanno et al. (2005), which was implemented in the STRUCTURE HARVESTER web version 0.6.94 (Earl & vonHoldt,

2012). CLUMPP v1.1.2 (Jakobsson & Rosenberg, 2007) was used to calculate the mean of the permuted matrices. Results were illustrated with Origin 2018 (Northampton, MA, USA).

BayesAss v3.0.4 (Wilson & Rannala, 2003) was implemented to calculate recent gene flow by calculating the migration rate among 37 populations in East China cities at the city level based on the nuclear microsatellites marker, due to limitation of computing power. We used four chains of 500,000,000 steps each with a burn-in of 100,000,000 steps with different seeds. The delta values for a, m, and f were 0.8, 0.5, and 0.9, respectively. Gelman-Rubin diagnostics in package coda (Plummer, Best, Cowles, & Vines, 2006) were used for convergence diagnosis in MCMC chains. In our analysis, every variable of MCMC chains had Gelman-Rubin potential scale reduction factors less than 1.1 and an effective sample size larger than 200.

To explore whether the city has a high dispersal potential for common ragweed in East China, we built a genetic graph. For the genetic graph, we constructed a directed weighted population graph of 15 metapopulations. The migration rates calculated by BayesAss v3.0.4 (Wilson & Rannala, 2003) were used to represent connectivity among sample cities. In this graph, the threshold m = 0.01 was applied to reduce the edge, producing a minimal directed graph (Dale & Fortin, 2010). The R packages POPGRAPH (Dyer, 2009) and IGRAPH (Csardi & Nepusz, 2006) were used to construct the graph and calculate graph centrality measures. The *Strength* centrality measure was calculated in this study. In our genetic graph, the *Strength* is an index of the magnitude of genetic exchange with all nodes connected to a given node.

Compare the similarity of common ragweed populations in East China cities to those in other countries

To identify the source populations of common ragweed populations in East China cities, each plant was assigned to French and Italian, modern North American, and historical North American populations. The North American samples were grouped by the genetic clusters identified by Martin et al. (2014). Bayesian criteria for likelihood estimation (Rannala & Mountain, 1997) were used because they performed better in the previous common ragweed assignment test (Genton, Shykoff, & Giraud, 2005). These tests were performed in GENECLASS2 (Piry et al., 2004) with an assignment threshold of 0.05 and the simulation algorithm of Paetkau et al. (2004) with 10000 simulated individuals from Monte-Carlo resampling based on the nuclear microsatellites markers.

Genetic diversity indices (including N_A, H_E, and F_{IS}) of French and Italian, and modern North American populations were estimated in GenAlEx 6.51 (Peakall & Smouse, 2012) and GENEPOP 4.7.0 (Rousset, 2008), respectively. Deviations from Hardy-Weinberg equilibrium were assessed across loci and sampling localities by F_{IS} (Weir & Cockerham, 1984), and significance was tested by 10000 Markov chains, 20 batches, and 5000 iterations per batch implemented in GENEPOP 4.7.0 (Rousset, 2008). An analysis of variance (ANOVA) implemented in the stats (R Core Team, 2018) package was used to test differences among the geographic ranges in H_E and F_{IS}.

Supplementary Figure 1. Null allele frequencies per locus.

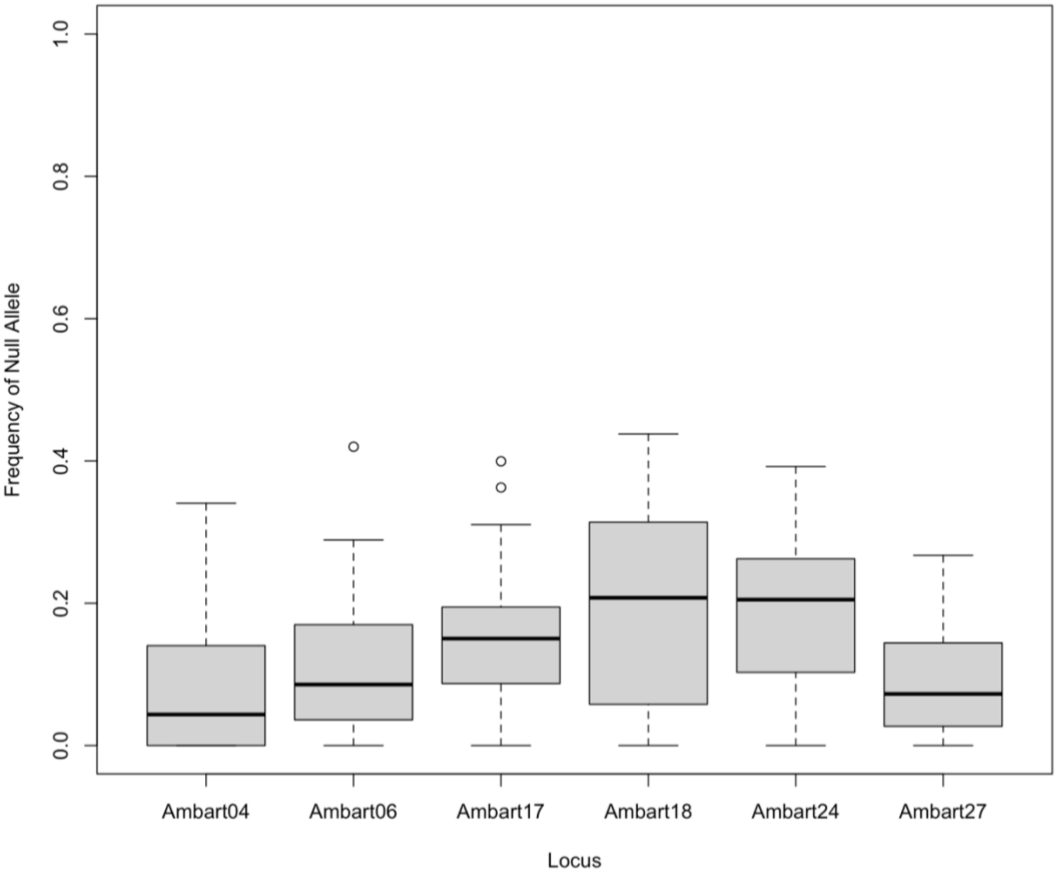

Y-axis presented the estimated percent of null alleles at each locus across all collected samples. Horizontal black bars represent the sample median. Box limits indicate upper- and lower-quartiles. The white point indicated the outliner.

Supplementary Figure 2. Phylogenetic relationship of cpDNA haplotype.

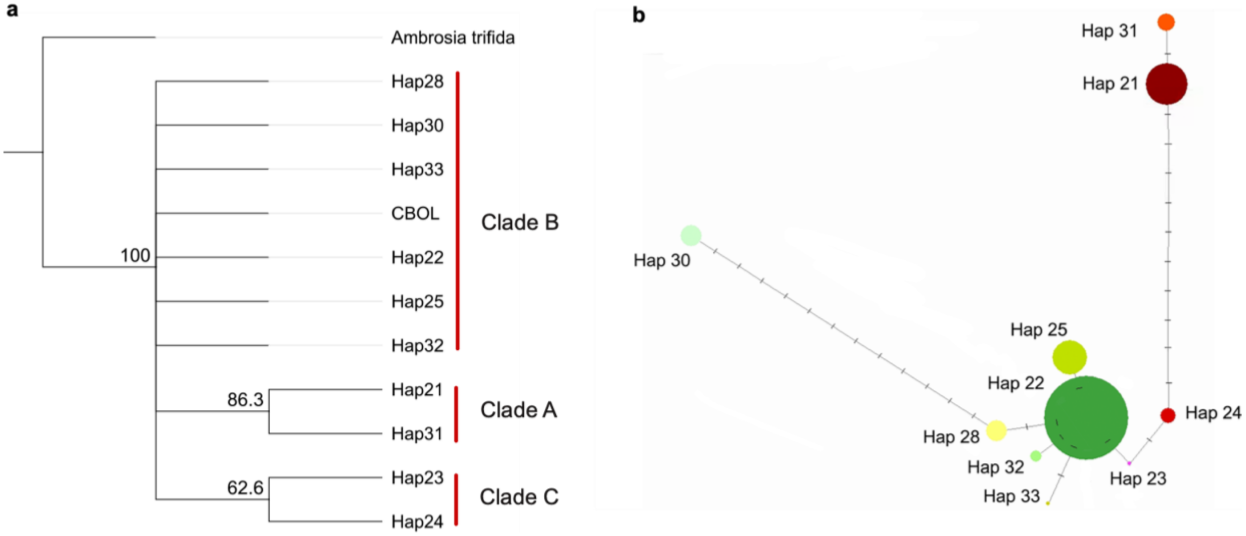

a. Rooted neighbor-Joining tree based on phylogenetic analyses of cpDNA (length = 802 bp). Bootstrap values are shown at nodes. b. Evolutionary paths of cpDNA haplotype in sequenced plants, circle size represented the number of plants of specific haplotype, color of the circle indicated the haplotype as in Figure 1, and branch length indicated the mutation distance.

Supplementary Figure 3.Results of GENELAND runs of urban populations in East China cities.

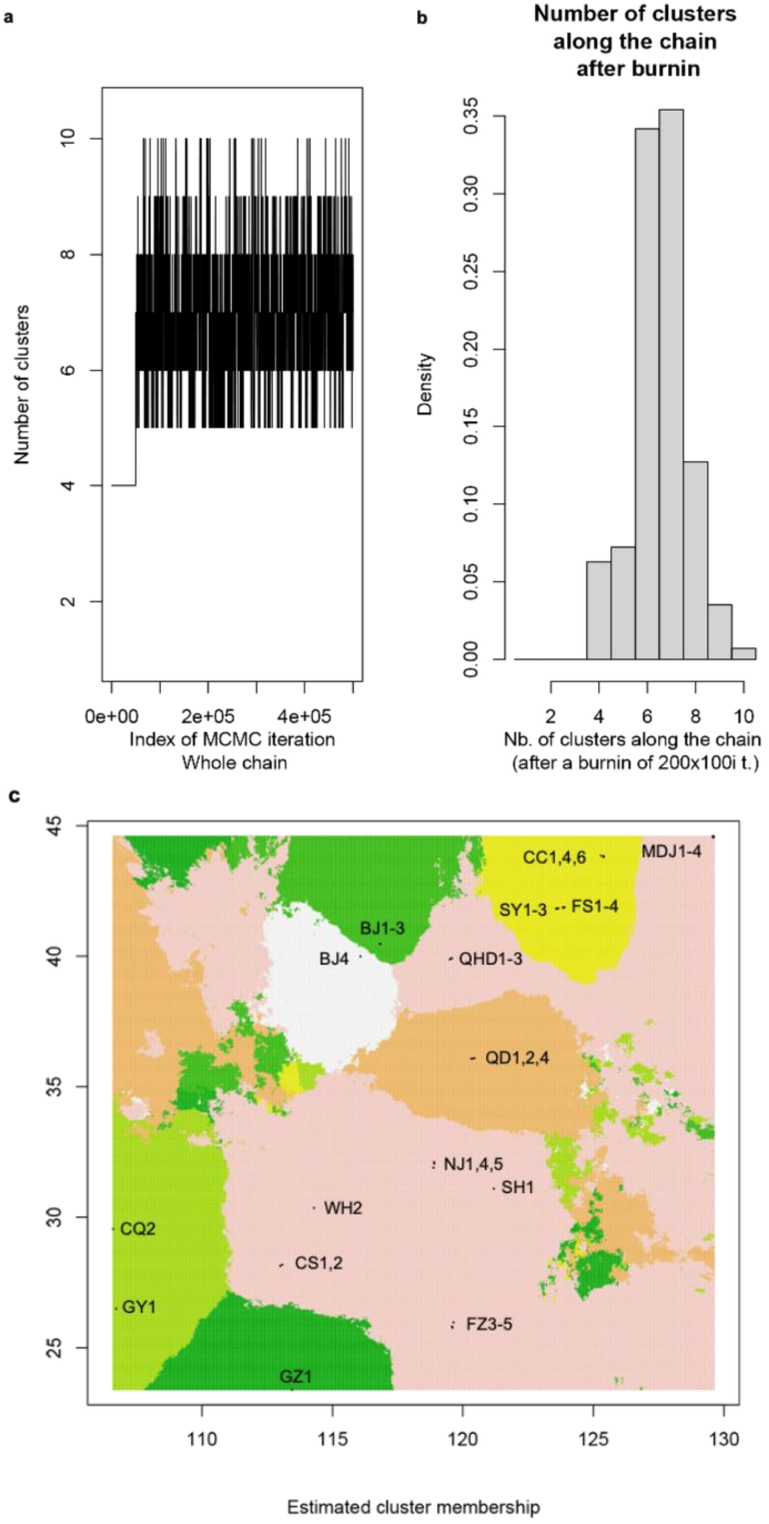

a. timeline of posterior density of the number of clusters. b. a histogram of the density of the number of clusters. c. GENELAND, k = 7. Using the spatial model with uncorrelated allele frequencies, map of estimated posterior probability of common ragweed population membership (by posterior mode). Abbreviations are names of sampled cities and populations: Mudanjiang (MDJ), Changchun (CC), Shenyang (SY), Fushun (FS), Qinhuangdao (QHD), Beijing (BJ), Qingdao (QD), Shanghai (SH), Nanjing (NJ), Wuhan (WH), Changsha (CS), Fuzhou (FZ), Guangzhou (GZ), Chongqing (CQ), Guiyang (GY).

Supplementary Figure 4.Likelihood results of STRUCTURE runs of urban populations in East China cities without a spatial algorithm with K = 1–10.

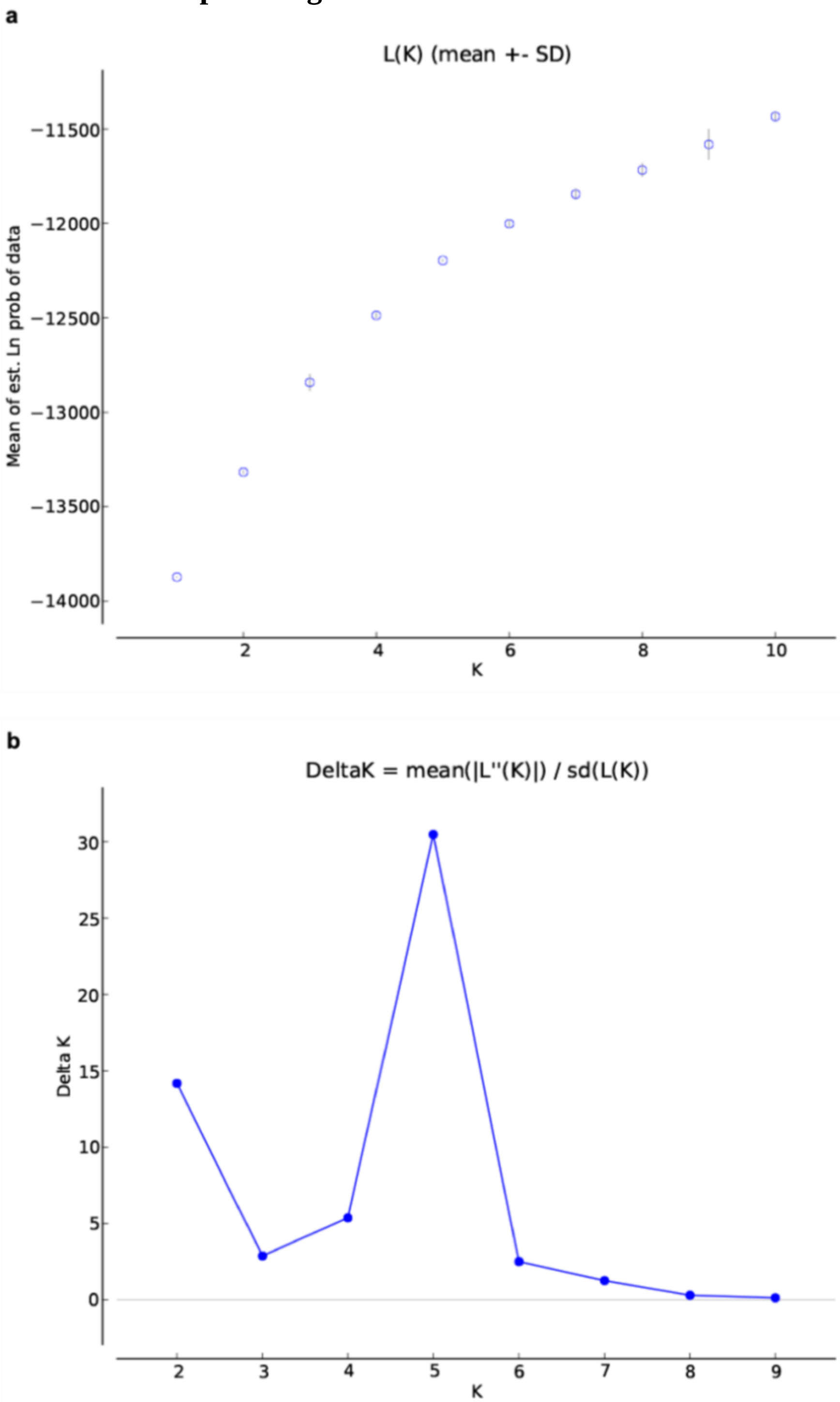

a. the mean log-likelihood ± SD of urban populations in East China cities. b. ΔK values of urban populations in East China cities.

Supplementary Figure 5.Heatmap of the F_{ST} matrix.

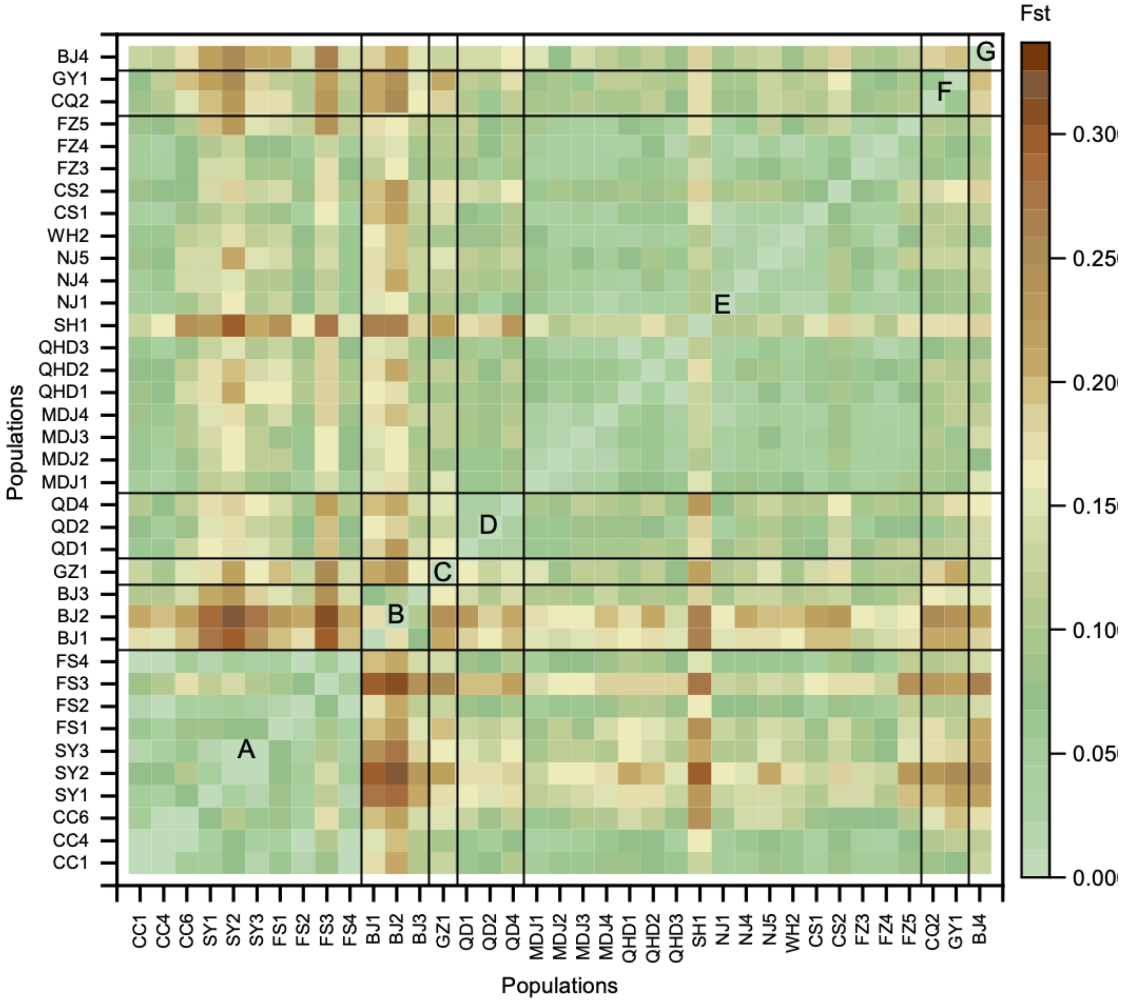

Gradation of color indicated incremental F_{ST}; 0 – 0.05 indicated moderate differentiation; 0.05 – 0.25 indicated notable differentiation; >0.25 indicated significant differentiation (Freeland, Kirk, & Petersen, 2011). A-G indicate the genetic clusters inferred by GENELAND. Abbreviations are names of sampled cities and populations: Mudanjiang (MDJ), Changchun (CC), Shenyang (SY), Fushun (FS), Qinhuangdao (QHD), Beijing (BJ), Qingdao (QD), Shanghai (SH), Nanjing (NJ), Wuhan (WH), Changsha (CS), Fuzhou (FZ), Guangzhou (GZ), Chongqing (CQ), Guiyang (GY).

Supplementary Figure 6. Comparison of expected heterozygosity and inbreeding coefficient among different regions.

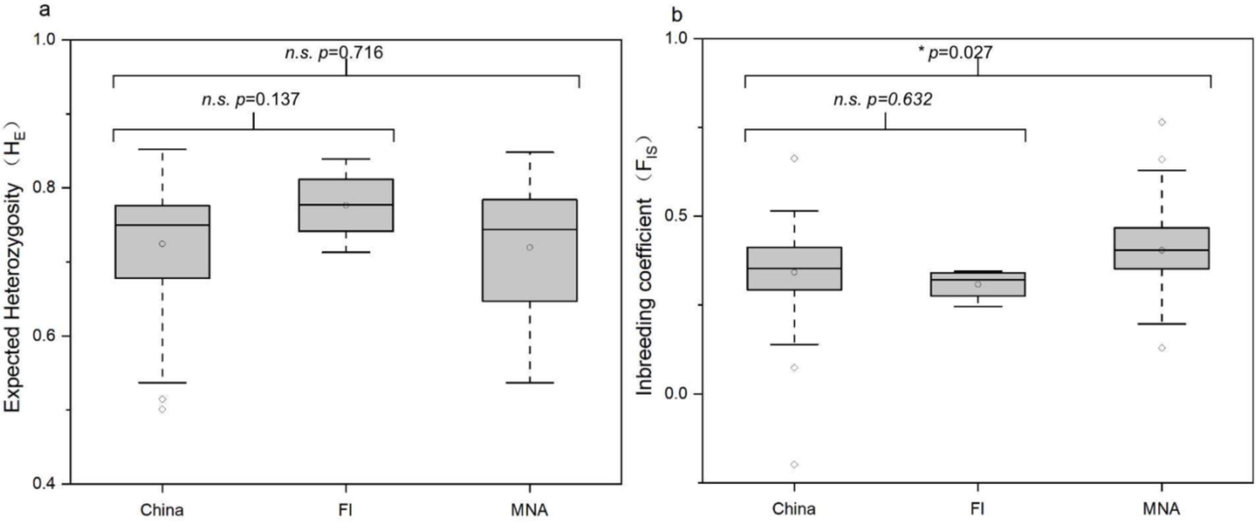

a. expected heterozygosity and **b.** inbreeding coefficient. Horizontal black bars represent the median value. Box limits indicate 75% and 25% quartiles. The white round point indicated the mean. The white diamond point indicated the outliner. boxes with the same letter mean they did not differ significantly ($p \leq 0.05$) based on the ANOVA. The sample size for China, FI and MNA is 55, 4, and 46, respectively. Abbreviation: FI (France and Italy), MNA (modern North America).

Supplementary Figure. 7. Sampling cities in East China.

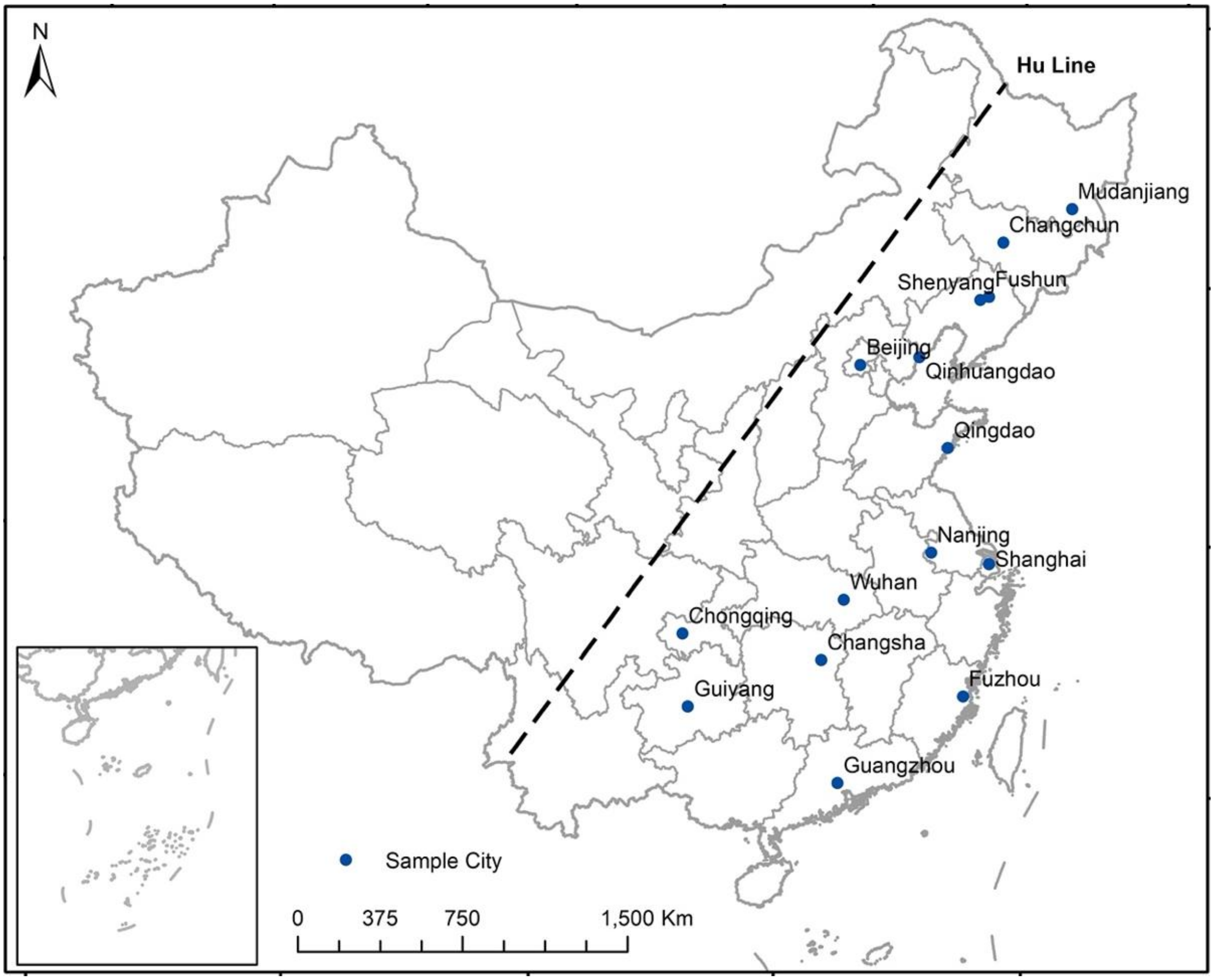

The dot line indicated the Hu Line.

Supplementary Table 1.

Location and grouping information of the urban populations in East China cities. Cluster, the clusters identified by GENELAND.

Population	Location	Latitude (°)	Longitude (°)	Cluster
BJ1	Beijing	40.47	116.84	B
BJ2	Beijing	40.48	116.82	B
BJ3	Beijing	40.48	116.81	B
BJ4	Beijing	39.99	116.06	G
CC1	Changchun	43.84	125.41	A
CC4	Changchun	43.87	125.31	A
CC6	Changchun	43.78	125.49	A
CQ2	Chongqing	29.54	106.6	F
CS1	Changsha	28.14	112.99	E
CS2	Changsha	28.18	113.07	E
FS1	Fushun	41.88	123.85	A
FS2	Fushun	41.88	123.9	A
FS3	Fushun	41.86	123.9	A
FS4	Fushun	41.86	123.83	A
FZ3	Fuzhou	25.99	119.7	E
FZ4	Fuzhou	25.78	119.61	E
FZ5	Fuzhou	25.81	119.61	E
GY1	Guiyang	26.49	106.72	F
GZ1	Guangzhou	23.39	113.45	C
MDJ1	Mudanjiang	44.6	129.57	E
MDJ2	Mudanjiang	44.58	129.57	E
MDJ3	Mudanjiang	44.56	129.59	E
MDJ4	Mudanjiang	44.59	129.62	E
NJ1	Nanjing	31.9	118.88	E
NJ4	Nanjing	32.06	118.91	E
NJ5	Nanjing	32.12	118.91	E
QD1	Qingdao	36.07	120.35	E
QD2	Qingdao	36.09	120.44	D
QD4	Qingdao	36.08	120.33	D
QHD1	Qinhuangdao	39.93	119.58	E
QHD2	Qinhuangdao	39.89	119.53	E
QHD3	Qinhuangdao	39.93	119.6	E
SH1	Shanghai	31.09	121.2	E
SY1	Shenyang	41.82	123.59	A

SY2	Shenyang	41.83	123.63	A
SY3	Shenyang	41.83	123.66	A
WH2	Wuhan	30.36	114.29	E

Supplementary Table 2.

Gene flow among 15 sampling cities. The gene flow migrated from column to row, gradation of color indicated the incremental migration rate.

	Beijing	Changchun	Chongqing	Changsha	Fushun	Fuzhou	Guiyang	Guangzhou	Mudanjiang	Nanjing	Qingdao	Qinhuangdao	Shanghai	Shenyang	Wuhan
Beijing		0.0045	0.0044	0.0045	0.0063	0.0049	0.0045	0.0044	0.0066	0.0062	0.0064	0.0048	0.0045	0.0049	0.0045
Changchun	0.0146		0.0073	0.0072	0.0411	0.0109	0.0073	0.0073	0.0234	0.0146	0.0152	0.0081	0.0073	0.1544	0.0072
Chongqing	0.0146	0.0146		0.0146	0.0201	0.0166	0.0146	0.0146	0.0473	0.0518	0.0353	0.0302	0.0146	0.0154	0.0145
Changsha	0.0099	0.0098	0.0098		0.0141	0.0109	0.0099	0.0099	0.0137	0.1721	0.0104	0.0133	0.0098	0.0201	0.0098
Fushun	0.0075	0.0049	0.0049	0.0049		0.0057	0.0049	0.005	0.0111	0.0172	0.0097	0.0057	0.0049	0.0492	0.005
Fuzhou	0.0153	0.0068	0.0068	0.0069	0.0194		0.0067	0.0069	0.0165	0.06	0.0123	0.0082	0.0068	0.0083	0.0069
Guiyang	0.0119	0.0111	0.0112	0.0111	0.0383	0.0137		0.0112	0.0138	0.0333	0.1149	0.0181	0.0111	0.0114	0.0111
Guangzhou	0.0133	0.0133	0.0134	0.0133	0.0133	0.1461	0.0134		0.0133	0.0134	0.0133	0.0137	0.0133	0.0135	0.0133
Mudanjiang	0.0069	0.0044	0.0043	0.0044	0.0102	0.006	0.0044	0.0044		0.0114	0.0074	0.0072	0.0043	0.005	0.0044
Nanjing	0.0067	0.006	0.006	0.0059	0.0098	0.0088	0.0059	0.006	0.0137		0.0091	0.0114	0.006	0.0113	0.006
Qingdao	0.0068	0.0065	0.0064	0.0065	0.0079	0.0067	0.0064	0.0064	0.0098	0.0087		0.0079	0.0064	0.0068	0.0064
Qinhuangdao	0.0083	0.0071	0.007	0.0071	0.0099	0.0165	0.007	0.007	0.0202	0.0278	0.0083		0.007	0.0101	0.007
Shanghai	0.0153	0.0152	0.0152	0.0151	0.0156	0.017	0.0151	0.0151	0.0441	0.0889	0.0151	0.0157		0.0155	0.0151
Shenyang	0.0076	0.006	0.0061	0.006	0.0151	0.0091	0.0061	0.0061	0.0083	0.0112	0.0066	0.0087	0.006		0.0061
Wuhan	0.0133	0.0104	0.0104	0.0104	0.0135	0.0179	0.0105	0.0104	0.0143	0.1651	0.0111	0.0144	0.0104	0.0105	

Supplementary Table 3

China's imports of grain products from 1935 to 1939. (Source: *Chinese Maritime Customs Historical Material: 1859-1948*).

Source country	Year					Sum
	1935 (U.S. Dollar)	1936 (U.S. Dollar)	1937 (U.S. Dollar)	1938 (U.S. Dollar)	1939 (U.S. Dollar)	(U.S. Dollar)
Australia	11,398,014	3,456,242	2,643,531	7,094,226	19,127,021	43,719,033
French indo-China	20,700,676	3,135,386	6,751,579	4,607,435	4,877,521	40,072,596
Thailand	8,803,473	4,682,929	3,891,981	7,208,332	6,077,587	30,664,302
Kwangchowwan leased territory	59,624	147,626	51,752	4,916,174	15,141,515	20,316,690
The U.S.	959,642	242,229	522,031	1,164,726	12,184,008	15,072,635
Japan	346,903	465,242	314,619	8,286,303	3,352,932	12,765,999
Burma	5,965,947	744,895	1,838,527	3,080,448	819,090	12,448,908
Korea	82,498	46,115	4,703	769,318	4,602,420	5,505,054
Canada	661,197	647,582	496,465	483,236	468,685	2,757,165
Argentina	2,538,624	0	0	0	0	2,538,624

Supplementary Table 4.

Nuclear microsatellite loci primer sequences and annealing temperatures.

Primer	Sequence	Annealing temp. (°C)	Fragment size range (bp)
Ambart04-F	5'-AGG GGT TAG TTC TCT TAG CAT C3'	53	143–232
Ambart04-R	5'-TCC GCG TGA TTG AGT TTA TG-3'		
Ambart06-F	5'-AAA GTG CCA CAA ACA CCT TG-3'	62	77–138
Ambart06-R	5'-CTG CCA AAA CAA CTG GAA GG-3'		
Ambart17-F	5'-GCC ATG GTA ATA CTA TCC TTA CAA CA3'	53	59–187
Ambart17-R	5'-TCA AGG ACC ACT GGG ACA TA3'		
Ambart18-F	5'-TGG TGG TGG AGC TTT TTG A3'	54	61–154
Ambart18-R	5'-AGC ACC TCA CGT TCC TCT T3'		
Ambart24-F	5'-GAT CAT TGC TGC AGT TTC AA3'	58	74-163
Ambart24-R	5'-CAT CAT TCA AAT TTT CCA TAT TG3'		
Ambart27-F	5'-CCC TTC CCT TCT GCT TC3'	61	143-227
Ambart27-R	5'-GCC ATC AAA ACA ACT ATG AAC 3'		

Supplementary Table 5.

Intergenic chloroplast spacer region primer sequences.

Region amplified	Primer pair	Primer sequence	Source
atpH–atpF spacer	atpH-F<br>atpH-R	5'-ACT CGC ACA CAC TCC CTT TC3'<br>5'-GCT TTT ATG GAA GCT TTA ACA AT-3'	CBOL 2009
psbK–psbI spacer	psbK-F<br>psbK-R	5'-TTA GCC TTT GTT TGG CAA G-3'<br>5'-AGA GTT TGA GAG TAA GCAT-3'	CBOL 2009

Supplementary Table 6.

Summary statistic of French and Italian, and modern North American populations. Bold values of F_{IS} indicate significant (p< 0.05) deviations from Hardy-Weinberg equilibrium. n, number of individuals sampled. N_A, number of different alleles. H_E, expected heterozygosity. F_{IS}, inbreeding coefficient.

Population	Location	Latitude (°)	Longitude (°)	n	N_A	H_E	F_{IS}
E-FRA3	Grane, France	44.75	4.88	12	10.333	0.839	**0.246**
E-PU	Pesaro, Italy	43.69	12.8	18	8.833	0.713	**0.337**
E-TO	Torino, Italy	45.15	7.75	12	7.833	0.784	**0.345**
E-TV	Treviso, Italy	45.77	12.33	12	8	0.77	**0.305**
U-AR	Arizona, USA	33.842	-93.776	16	9.833	0.749	**0.424**
U-CAN3	Quebec, Canada	45.16	-73.67	12	8.833	0.791	**0.292**
U-CTE	Connecticut, USA	41.981	-72.077	8	6.667	0.76	**0.27**
U-CTG	Connecticut, USA	41.78	-73.717	8	8.667	0.848	**0.432**
U-FLA	Florida, USA	25.542	-80.413	8	5.667	0.716	**0.44**
U-FLD	Florida, USA	27.374	-80.416	7	5.333	0.73	**0.328**
U-FLG	Florida, USA	27.085	-81.795	15	6.833	0.696	**0.358**
U-GA	Georgia, USA	31.679	-83.196	16	8	0.798	**0.495**
U-IA	Iowa, USA	41.875	-93.483	10	5	0.649	**0.765**
U-IL	Illinois, USA	39.238	-88.151	12	6.667	0.671	**0.4**
U-LA	Louisiana, USA	30.469	-90.643	19	8.833	0.768	**0.495**
U-MAA	Massachusetts, USA	42.311	-71.161	8	6.333	0.792	**0.371**
U-MAB	Massachusetts, USA	41.785	-71.078	8	4.667	0.693	**0.392**
U-MAD	Massachusetts, USA	41.947	-70.784	8	4.333	0.627	0.129
U-MAE	Massachusetts, USA	42.287	-72.003	12	8.667	0.814	**0.468**
U-MAF	Massachusetts, USA	42.318	-72.426	7	6.667	0.755	**0.232**
U-MAG	Massachusetts, USA	42.651	-70.794	8	7.333	0.812	**0.381**
U-MEB	Maine, USA	44.632	-69.508	8	5.833	0.789	**0.463**
U-MEC	Maine, USA	44.013	-70.069	8	6.167	0.784	**0.485**
U-MED	Maine, USA	44.967	-68.72	9	6.5	0.783	**0.547**
U-MEE	Maine, USA	45.158	-67.289	8	4.5	0.641	**0.241**
U-MIA	Michigan, USA	46.172	-84.365	12	5.167	0.628	**0.482**
U-MIB	Michigan, USA	43.154	-84.504	10	6.667	0.603	**0.352**
U-MN	Minnesota, USA	45.794	-94.245	10	3.5	0.568	**0.63**
U-MOA	Missouri, USA	38.968	-93.879	11	4.667	0.617	**0.66**
U-MOB	Missouri, USA	38.386	-90.259	8	6.167	0.778	**0.418**
U-MOC	Missouri, USA	39.709	-91.5	8	6.333	0.767	**0.485**
U-NHA	New Hampshire, USA	42.891	-72.528	8	7	0.783	**0.422**
U-NHB	New Hampshire, USA	42.929	-70.992	8	6.333	0.739	**0.332**

U-NHC	New Hampshire, USA	42.812	-71.591	7	5.5	0.686	**0.46**
U-NJA	New Jersey, USA	40.74	-74.953	8	7.5	0.79	**0.456**
U-NJB	New Jersey, USA	40.504	-74.742	8	6.667	0.806	**0.366**
U-NJD	New Jersey, USA	39.149	-74.851	8	7.833	0.8	**0.406**
U-NJF	New Jersey, USA	40.226	-74.258	9	7	0.674	**0.381**
U-NJI	New Jersey, USA	41.629	-74.182	10	7.667	0.623	**0.369**
U-NSA	Nova Scotia, Canada	44.909	-65.177	8	5.667	0.729	**0.502**
U-NSB	Nova Scotia, Canada	44.755	-63.672	7	6	0.709	**0.238**
U-NSC	Nova Scotia, Canada	43.874	-64.967	23	7	0.647	**0.386**
U-OHB	Ohio, USA	38.91	-82.728	9	4	0.585	**0.436**
U-PAA	Pennsylvania, USA	40.079	-79.615	10	5.333	0.537	**0.458**
U-RIA	Rhode Island, USA	41.683	-71.134	10	5.167	0.604	**0.403**
U-RIB	Rhode Island, USA	41.485	-71.558	8	6.167	0.772	**0.231**
U-RIC	Rhode Island, USA	41.794	-71.534	8	7	0.816	**0.385**
U-SC	South Carolina, USA	34.793	-80.415	15	8.833	0.765	**0.197**
U-TN	Tennessee, USA	36.119	-85.059	14	7.667	0.768	**0.257**
U-WI	Wisconsin, USA	45.415	-89.19	14	7.5	0.643	**0.503**
