# Supplementary material for: China-US grain trade shapes the spatial genetic pattern of common ragweed in East China cities
Source: Commun Biol. 2023 Oct 21;6:1072. doi: 10.1038/s42003-023-05434-5 (PMC10590438; doi:10.1038/s42003-023-05434-5)
Supplement: Supplementary file 2 — Reporting Summary [file 42003_2023_5434_MOESM2_ESM.pdf]

nature portfolio

Corresponding author(s): Jun Yang

Last updated by author(s): Oct 4, 2023

Reporting Summary

Nature Portfolio wishes to improve the reproducibility of the work that we publish. This form provides structure for consistency and transparency in reporting. For further information on Nature Portfolio policies, see our Editorial Policies and the Editorial Policy Checklist.

Statistics

For all statistical analyses, confirm that the following items are present in the figure legend, table legend, main text, or Methods section.

n/a	Confirmed	
☐	☒	The exact sample size (*n*) for each experimental group/condition, given as a discrete number and unit of measurement
☐	☒	A statement on whether measurements were taken from distinct samples or whether the same sample was measured repeatedly
☒	☐	The statistical test(s) used AND whether they are one- or two-sided<br>*Only common tests should be described solely by name; describe more complex techniques in the Methods section.*
☒	☐	A description of all covariates tested
☒	☐	A description of any assumptions or corrections, such as tests of normality and adjustment for multiple comparisons
☐	☒	A full description of the statistical parameters including central tendency (e.g. means) or other basic estimates (e.g. regression coefficient) AND variation (e.g. standard deviation) or associated estimates of uncertainty (e.g. confidence intervals)
☐	☒	For null hypothesis testing, the test statistic (e.g. *F*, *t*, *r*) with confidence intervals, effect sizes, degrees of freedom and *P* value noted<br>*Give P values as exact values whenever suitable.*
☐	☒	For Bayesian analysis, information on the choice of priors and Markov chain Monte Carlo settings
☒	☐	For hierarchical and complex designs, identification of the appropriate level for tests and full reporting of outcomes
☒	☐	Estimates of effect sizes (e.g. Cohen's *d*, Pearson's *r*), indicating how they were calculated

Our web collection on statistics for biologists contains articles on many of the points above.

Software and code

Policy information about availability of computer code

Data collection	DNA extraction, amplification, and sequencing was performed on Applied Biosystems of Thermo Fisher (Sanger method).<br>R 4.0.2 was used to work up raw grain importation data, R code can be found through the Mendeley Data with identifier DOI:10.17632/kxgtk8r5kz.1.
Data analysis	The following computer softwares were used for data analysis:<br>MAFFT,<br>GENEIOUS V11.0.4,<br>GenAlEx 6.51,<br>GENEPOP 4.7.0,<br>BOTTLENECK 1.2.0.2,<br>DnaSP 6,<br>STRUCTURE 2.3.3,<br>GENELAND 4.0.3,<br>STRUCTURE HARVESTER web version 0.6.94,<br>CLUMPP v1.1.2,<br>Origin 2018,<br>ArcGIS desktop version 10.2,<br>NETWORK 10.1,<br>BayesAss v3.0.4,<br>GENECLASS2,<br>R package diveRsity,

R package POPGRAPH,
R package IGRAPH,
R 4.0.2

For manuscripts utilizing custom algorithms or software that are central to the research but not yet described in published literature, software must be made available to editors and reviewers. We strongly encourage code deposition in a community repository (e.g. GitHub). See the Nature Portfolio guidelines for submitting code & software for further information.

Data

Policy information about availability of data

All manuscripts must include a data availability statement. This statement should provide the following information, where applicable:

- Accession codes, unique identifiers, or web links for publicly available datasets
- A description of any restrictions on data availability
- For clinical datasets or third party data, please ensure that the statement adheres to our policy

Microsatellite genotypes, chloroplast haplotype, input files and R code used for population genetic, occurrence data, grain exportation and importation data analysis are available in the Mendeley Data with identifier DOI:10.17632/kxgtk8r5kz.1. Chloroplast intergenic spacer locus sequences that support the findings of this study have been deposited in GenBank with the accession numbers from MZ826750 to MZ826763.

Research involving human participants, their data, or biological material

Policy information about studies with human participants or human data. See also policy information about sex, gender (identity/presentation), and sexual orientation and race, ethnicity and racism.

Reporting on sex and gender	none
Reporting on race, ethnicity, or other socially relevant groupings	none
Population characteristics	none
Recruitment	none
Ethics oversight	none

Note that full information on the approval of the study protocol must also be provided in the manuscript.

Field-specific reporting

Please select the one below that is the best fit for your research. If you are not sure, read the appropriate sections before making your selection.

☐ Life sciences ☐ Behavioural & social sciences ☒ Ecological, evolutionary & environmental sciences

For a reference copy of the document with all sections, see nature.com/documents/nr-reporting-summary-flat.pdf

Ecological, evolutionary & environmental sciences study design

All studies must disclose on these points even when the disclosure is negative.

Study description	We sampled common ragweed populations in 15 East China cities to quantified the spatial genetic pattern and inferred invasion sources of urban populations. We also inferred city had a high dispersal potential.
Research sample	We sampled in the urban area of East China cities. East China is the suitable habitat for the invasion of common ragweed and holds most of the Chinese population. We also collected genetic data of native North American populations from Martin et al. (2014) and data of invasive French and Italian populations from Ciappetta et al. (2016).
Sampling strategy	We sampled 37 common ragweed populations in 15 East China cities. Limited to varied population size of urban populations, 6 to 19 plants were sampled at least 2 m from each other inside each population. We sampled at least 5 fresh leaves for each plant and stored leaves in sealed plastic bags filled with silica beads for desiccation.
Data collection	DNA extraction, amplification, and sequencing was performed on Applied Biosystems of Thermo Fisher (Sanger method) by Bio-ulab company (Beijing, China) in 2017 and 2018. Detail of methods is described in supplementary material.
Timing and spatial scale	To better discriminate common ragweed plants, we collected samples in flowering season of common ragweed (Sept. 2017 to Oct 2017 and Sept. 2018 to Oct 2018). Changchun, Qinhuangdao, Qingdao and Changsha were sampled in 2017. Mudanjiang, Shenyang, Fushun, Beijing, Nanjing, Shanghai, Fuzhou, Wuhan, Guangzhou, Chongqing and Guiyang were sampled in 2018.

Data exclusions	In the analysis of cpDNA haplotypes, all plants from population CS2 were excluded for we can't sequence their chloroplast DNA. some plants from other populations were excluded for the same reason (detail of exclusion can be found in Table 1)
Reproducibility	Reproducibility is generally not feasible for population genetic study
Randomization	Randomization is generally not feasible for population genetic study
Blinding	Blinding is generally not feasible for population genetic study

Did the study involve field work? ☒ Yes ☐ No

Field work, collection and transport

Field conditions	There is no need to consider the weather condition.
Location	Sampling location information can be found in supplementary material Table S1.
Access & import/export	None
Disturbance	None

Reporting for specific materials, systems and methods

We require information from authors about some types of materials, experimental systems and methods used in many studies. Here, indicate whether each material, system or method listed is relevant to your study. If you are not sure if a list item applies to your research, read the appropriate section before selecting a response.

Materials & experimental systems

n/a	Involved in the study	
☒	☐	Antibodies
☒	☐	Eukaryotic cell lines
☒	☐	Palaeontology and archaeology
☒	☐	Animals and other organisms
☒	☐	Clinical data
☒	☐	Dual use research of concern
☐	☒	Plants

Methods

n/a	Involved in the study	
☒	☐	ChIP-seq
☒	☐	Flow cytometry
☒	☐	MRI-based neuroimaging

Dual use research of concern

Policy information about dual use research of concern

Hazards

Could the accidental, deliberate or reckless misuse of agents or technologies generated in the work, or the application of information presented in the manuscript, pose a threat to:

No	Yes	
☒	☐	Public health
☒	☐	National security
☒	☐	Crops and/or livestock
☒	☐	Ecosystems
☒	☐	Any other significant area

Experiments of concern

Does the work involve any of these experiments of concern:

No	Yes	
☒	☐	Demonstrate how to render a vaccine ineffective
☒	☐	Confer resistance to therapeutically useful antibiotics or antiviral agents
☒	☐	Enhance the virulence of a pathogen or render a nonpathogen virulent
☒	☐	Increase transmissibility of a pathogen
☒	☐	Alter the host range of a pathogen
☒	☐	Enable evasion of diagnostic/detection modalities
☒	☐	Enable the weaponization of a biological agent or toxin
☒	☐	Any other potentially harmful combination of experiments and agents
